# Child and adolescent mental health and psychosocial support interventions: An evidence and gap map of low‐ and middle‐income countries

**DOI:** 10.1002/cl2.1349

**Published:** 2023-08-23

**Authors:** Ruichuan Yu, Camila Perera, Manasi Sharma, Alessandra Ipince, Shivit Bakrania, Farhad Shokraneh, Juan Sebastian Mosquera Sepulveda, David Anthony

**Affiliations:** ^1^ UNICEF Innocenti—Global Office of Research and Foresight, UNICEF HQ Florence Italy

## Abstract

**Background:**

Mental disorders affect about one in seven children and adolescents worldwide. Investment in effective child and adolescent mental health prevention, promotion and care is essential. To date, however, the evidence from this field is yet to be comprehensively collected and mapped.

**Objectives:**

The objective of this evidence and gap map (EGM) is to provide an overview of the existing evidence on the effectiveness of interventions aimed at promoting mental health and reducing or preventing mental health conditions among children and adolescents in lower‐middle‐income countries (LMICs).

**Search Methods:**

We searched for studies from a wide range of bibliographic databases, libraries and websites. All searches were conducted in December 2021 and covered the period between 2010 and 2021.

**Selection Criteria:**

We included evidence on the effectiveness of any Mental Health and Psychosocial Support (MHPSS) interventions targeting children and adolescents from 0 to 19 years of age in LMICs. The map includes systematic reviews and effectiveness studies in the form of randomised control trials and quasi‐experimental studies, and mixed‐methods studies with a focus on intervention effectiveness.

**Data Collection and Analysis:**

A total of 63,947 records were identified after the search. A total of 19,578 records were removed using machine learning. A total of 7545 records were screened independently and simultaneously by four reviewers based on title and abstract and 2721 full texts were assessed for eligibility. The EGM includes 697 studies and reviews that covered 78 LMICs.

**Main Results:**

School‐based interventions make up 61% of intervention research on child and adolescent mental health and psychosocial support. Most interventions (59%) focusing on treating mental health conditions rather than preventing them or promoting mental health. Depression (40%, *N* = 282) was the most frequently researched outcome sub‐domain analysed by studies and reviews, followed by anxiety disorders (32%, *N* = 225), well‐being (21%, *N* = 143), and post‐traumatic stress disorder (18%, *N* = 125). Most included studies and reviews investigated the effectiveness of mental health and psychosocial support interventions in early (75%, *N* = 525) and late adolescence (64%, *N* = 448).

**Conclusions:**

The body of evidence in this area is complex and it is expanding progressively. However, research on child and adolescent MHPSS interventions is more *reactive* than *proactive*, with most evidence focusing on addressing mental health conditions that have already arisen rather than preventing them or promoting mental health. Future research should investigate the effectiveness of digital mental health interventions for children and adolescents as well as interventions to address the mental health and psychosocial needs of children in humanitarian settings. Research on early childhood MHPSS interventions is urgently needed. MHPSS research for children and adolescents lacks diversity. Research is also needed to address geographical inequalities at the regional and national level. Important questions also remain on the quality of the available research—is child and adolescent MHPSS intervention research locally relevant, reliable, well‐designed and conducted, accessible and innovative? Planning research collaborations with decision‐makers and involving experts by experience in research is essential.

## 1 PLAIN LANGUAGE SUMMARY

### 1.1 **Evidence and gap map** (EGM) finds a stronger focus on treating than preventing mental health problems of children and adolescents

Most research on child and adolescent mental health and psychosocial interventions is reactive rather than proactive, with a strong focus on treating rather than preventing mental health problems or promoting mental health.

### 1.2 What is this EGM about?

Half of all mental health problems originate early in life. Many are preventable, but most remain unrecognised and untreated. Investment in effective child and adolescent mental health prevention, promotion and care is therefore essential.

This EGM provides a visual overview of the existing evidence on the effectiveness of mental health and psychosocial support interventions for children and adolescents aged 0–19 years in lower‐middle‐income countries (LMICs).

The interventions are divided into four categories: school‐based, community‐based, individual and family‐based, and digital. These are then further filtered by where, how, for what and to whom they are delivered.

**What is the aim of this EGM?**

This map visually presents evidence from 697 studies and reviews conducted between 2010 and 2021 on the effectiveness of mental health and psychosocial support interventions for children and adolescents in low‐ and middle‐income countries.

### 1.3 What studies are included?

A total of 697 studies and reviews are captured in the EGM, focusing on 78 LMICs.

### 1.4 What are the main findings of this EGM?

Most records cover lower‐middle‐income countries, with a few covering low‐income countries.

Most of the records examine the effectiveness of interventions among adolescents. Most interventions focus on treating mental health problems rather than preventing them or promoting mental health.

School‐based interventions are the most studied, followed by community‐based interventions, individual and family‐based interventions. Digital interventions are the least researched platform.

Most studies investigated mental health conditions, followed by mental health and early childhood development outcomes. Depression was the most frequently researched outcome sub‐domain, followed by anxiety disorders, well‐being, and post‐traumatic stress disorder.

### 1.5 What do the findings mean?

Research evidence on mental health and psychosocial support interventions for children and adolescents in LMICs is progressively expanding but unevenly distributed among regions and countries and by intervention and outcome domains.

Most of the evidence focuses on treating mental health disorders rather than preventing or enhancing mental health, indicating that current research is more reactive than proactive.

Mental health and psychosocial support research for children and adolescents lacks diversity. It is critical to include certain sub‐populations in studies, particularly those that tend to report a higher prevalence of mental health and psychosocial problems and are less likely to have access to mental health care. More evidence is needed on the effectiveness of digital mental health interventions, interventions in humanitarian settings, and interventions for the youngest children.

There are concerns about the quality of the available research. Progress on mental health and psychosocial support is hampered by a lack of investment in robust research on which interventions work to improve child and adolescent mental health.

### 1.6 How up‐to‐date is this EGM?

All the searches were conducted in December 2021, to retrieve all systematic reviews and primary studies published between January 2010 and December 2021, with no language restrictions.

## 2 BACKGROUND

### 2.1 The problem, condition or issue

All children have the right to survive, grow and develop, within the context of physical, emotional and social well‐being, to achieve their full potential (UN, 2013). Mental health has been defined as ‘a state of well‐being in which the individual realises his or her own abilities, can cope with the normal stresses of life, can work productively and fruitfully, and is able to make a contribution to his or her community’ (WHO, 2004, p. 11). While this definition moves away from the conceptualisation of mental health as solely the absence of illness, most research and prevalence studies on children and adolescents focus on the mental health conditions that affect mood, thinking and behaviour.

It is estimated that globally mental disorders affect about one in seven children and adolescents aged 10–19 (UNICEF, 2021). The magnitude and nature of child and adolescent mental health conditions can be illustrated through several key figures. First, and despite significant variation, the worldwide pooled prevalence of mental health conditions among children aged 10–19 is estimated at 27.5% for anxiety disorders and 12.7% for depression, which are often comorbid (UNICEF, 2021). Second, depression is among the leading causes of disability among young people while suicide is a leading cause of death among children and adolescents worldwide, ages 10–19 (UNICEF, 2021). Although behavioural problems among younger age groups are prevalent and vary in intensity, there is limited data on global rates and a limited understanding of their long‐term consequences (Hong, 2015). Lastly, most mental health conditions originate early in life, with 50% arising before the age of 14 and 75% by the mid‐20s (Kessler, 2010; Solmi, 2021). The evidence on effective interventions addressing the mental health and psychosocial well‐being of children and adolescents has not been consistently gathered and mapped, despite the prevalence of these conditions.

Across the phases of life, experiences and environment present potential risks and opportunities for children and adolescents. Mounting evidence has shown that the first 1000 days represent a unique opportunity for cognitive growth and early stimulation which are central to healthy mental and emotional lives (Erskine, 2017; Klasen & Crombag, 2013; Patel, 2018). During the early years of a child's life, parents and caregivers are instrumental in shaping child development and behaviour through adequate child nutrition, education and a nurturing and safe home environment. Middle childhood (5–9 years) are school‐going years that provide the context for early peer support and nurturing care through positive interactions as well as providing opportunities for building important life skills (Kieling, 2011).

Adolescence offers a second window of opportunity, representing a critical period in brain development where adolescents adopt and maintaining social and emotional habits and engage in identity formation. This period is characterised by a heightened salience of relationships with peers, as key to shaping and directing young people's psychological development (Mitic, 2021). The onset of puberty at this stage brings unique mental health challenges compounded by physiological and emotional transitions, as well as sexual and risk‐taking behaviours. Late adolescence (15–19 years) is shaped by community and social and cultural expectations of acceptable behaviour, gender norms and roles, and the upper end of adolescence comes with pressure to secure employment and gain social and economic independence.

Although most children can adequately recover and adapt using their own resources, childhood and adolescence are also vulnerable periods during which adverse experiences can negatively impact cognitive, emotional, and behavioural development. Without care and support, some children and adolescents can carry the mental and emotional costs of exposure to adverse experiences during earlier years for years to come (Haahr‐Pedersen, 2020). Indeed, research shows that in low‐resource settings, multiple, overlapping childhood adversities (e.g., violence, neglect, abuse, parental separation or substance use) are consistently associated with poor mental health (Jokinen, 2021; Kieling, 2011; Reed, 2012). At the same time, evidence highlights that adolescence is a time when young people harness skills and traits that can foster resilience, or the learned capacity to deal more effectively with ongoing adversity (Lansford, 2018). Effective positive coping strategies and behaviours adopted and learned during these years can reap benefits into adulthood. Throughout childhood and adolescence, children can be helped to develop resilience by, for example, helping parents to be more responsive to children's emotional and material needs, building community cohesion and providing children with high‐quality learning opportunities.

Despite the high burden and early onset, most mental health conditions remain unrecognised and untreated. A global systematic review of survey data in 2004 estimated that 70% of people aged 15 and older who were living with mental health conditions lacked access to adequate care (Kohn, 2004) and that this gap is higher in LMICs, where most (90%) children and young people live (Kieling, 2011). It is important to assess the evidence and treatment gaps in LMICs in more recent years, as this estimate may have changed in the last decade. Additionally, there is growing evidence of effective, affordable and culturally acceptable interventions from high‐income settings for preventing and treating mental health conditions that can be implemented in LMICs (Das, 2016). School‐based programmes can have significant positive effects on children and adolescents' well‐being, including reduced depression and anxiety and improved coping skills (Barry, 2013). Various promotion and prevention approaches have been successfully implemented and rolled‐out in community settings (Bradshaw, 2021; Das, 2016; Klasen & Crombag, 2013; Skeen, 2019). Parent and family‐focused interventions (i.e., psychoeducation, parent and family‐skills training, behavioural, psychosocial, and trauma‐focused cognitive behavioural therapy) may be beneficial to child and youth mental health and well‐being, as well as parenting behaviours and family functioning (Pedersen, 2019).

### 2.2 Why it is important to develop the EGM

Investment in child and adolescent mental health prevention, promotion and care is essential, but the evidence from this field is yet to be systematically collected and mapped. An EGM generates a clearer picture of the available evidence on interventions to improve child and adolescent mental health in low‐resource settings, thereby informing future research, policy and practice.

Promoting, protecting and caring for children and young people's mental health plays a key role in achieving all of the 17 sustainable development goals (SDGs). More specifically, Goal 3 calls on Member States to ensure healthy lives and promote well‐being for all *at all ages*. SDG target 3.4 aims to reduce premature mortality from non‐communicable diseases and to promote mental health and well‐being. Effective mental health interventions can act as potential development goal accelerators (Sherr, 2020)—with provisions that lead to progress across domains of a child's life and impacting upon multiple SDGs (Patel, 2018). However, mental health care is chronically under‐prioritised and under‐funded, representing just up to 1% of national health budgets in LMICs (Patel, 2018). In the context of meeting these global goals, there is an urgent need to identify *what works* in the field of mental health and psychosocial support in low‐resource settings and mapping potential areas of investment for future research and programming.

Early evidence from the COVID‐19 crisis indicates exacerbated mental health problems during the pandemic, with children and young people globally at risk of psychosocial distress, including anxiety, depression, and externalising behaviours, due to lockdowns, school closures and economic recession (UNICEF Office of Research—Innocenti, 2021). The need for greater investment in mental health interventions emerged as a key finding in a recent rapid review on the topic (UNICEF Office of Research—Innocenti, 2021), as well as the importance of targeting specific risk and protective factors across age groups in LMICs, where the research is most limited. A lack of understanding of what works acts as a barrier to increasing investment in mental health care (McDaid, 2008). More research is needed to examine the state of the evidence regarding programmatic interventions on children's mental health in the first and second decades of life. Yet, no unified resource exists that provides an overview of the evidence of child and adolescent mental health interventions in these settings.

Against this backdrop, UNICEF has renewed its commitment by setting a new goal to secure investment and action to support and protect the mental health of children and young people. It is estimated that 90% of research on child and adolescent mental health has been conducted in high‐income countries and evidence from low‐resource settings is sparse (Kieling, 2011). An EGM identifies where the evidence is abundant, but also where limited research and absolute gaps exist and increase the visibility of the available evidence (Saran, 2020). This resource enables us to identify under‐researched areas, countries and population sub‐groups and to inform the decisions of international donors, policymakers, practitioners, and researchers as well as UNICEF's research priorities and programmatic actions. A visual representation of the evidence, in the form of a matrix of interventions, allows practitioners, researchers, donors and policymakers to identify and focus on the areas of research that are more likely to inform their work.

### 2.3 Existing EGMs and/or relevant systematic reviews

A brief desk‐based scoping of the literature was conducted to inform the objectives of this EGM, which identified several EGMs covering adjacent topics and themes, and systematic reviews that explore a subset of the interventions and outcomes being proposed for inclusion in this EGM.

The *Mega Map of Child Well‐Being Interventions in Low‐ and Middle‐Income Countries* (Saran, 2020) includes evidence on parenting interventions and associated mental health and psychosocial well‐being outcomes that are relevant to the scope of our EGM, but does not map the evidence on mental health promotion, prevention and care interventio*ns. Another recent EGM on Interventions for reducing violenc*e against children in LMICs (Pundir, 2020) provides a valuable snapshot of the impact of violence prevention interventions on child mental health outcomes. However, it does not purposefully include interventions to address child and adolescent mental health and the outcomes are not explored to the same level of detail as is proposed here. In an EGM on *Adolescent Well‐Being in Low‐ and Middle‐Income Countries*, Bakrania (2018) gathered psychosocial support interventions but excluded mental health outcomes. In the context o*f the COVID‐19 pandemic, a Rapid Evidence and Gap Map of Virtual Care Solutions for Youth and Families to Mitigate the Impact of the COVID‐19 Pandemic on Pain, Menta*l Health, and Substance Use identified a series of virtual psychological interventions for management of chronic pain among young people during the pandemic (Birnie, 2021). Lastly, an EGM on interventions for children and adolescents with disabilities is currently underway (Thota, 2022) and being conducted under common supervision with this EGM to manage cross‐over areas and avoid duplication.

In addition to available and upcoming EGMs, a number of systematic reviews have investigated specific subsets of mental health interventions, outcomes and populations, and will be considered according to our inclusion and exclusion criteria. Barry (2013) reviewed the evidence on the effectiveness of mental health promotion interventions for young people (aged 6–18 years) in school and community settings and Clarke (2015) focused on online prevention interventions. Jordans (2009) synthesised research on interventions addressing child mental health and psychosocial well‐being in conflict settings. More recently, a systematic review assessed the evidence of mental health and psychosocial support programmes for children and adults affected by humanitarian emergencies (Bangpan, 2017) and Lloyd‐Reichling (2005) focused on younger children (0–8 years). Bradshaw (2021) reviewed the evidence on scalable school‐based interventions to prevent and address mental health concerns in LMICs. A systematic review identified psychosocial interventions that effectively promote positive mental health and prevent mental health conditions in pregnant and parenting adolescents (Laurenzi, 2020). A meta‐analysis identified effective programme components of interventions to promote mental health and prevent mental disorders and risk behaviours during adolescence (Skeen, 2019). In a review of systematic reviews, Das (2016) synthesised the evidence on mental health intervention for adolescents, including but not limited to virtual, individual, group, family and school‐based interventions. Another review investigated programmes aimed at promoting mental health and preventing mental disorders and risk behaviours during adolescence (Skeen, 2019). Klasen and Crombag (2013) identified a series of affordable and feasible interventions for children and adolescents in low‐resource settings. van Ginneken (2013, 2021), respectively analysed the effectiveness of non‐specialist and primarly level worker mental health and psychosocial support interventions on child and adolescent mental health.

We also identified and reviewed relevant intervention guidelines and their supporting evidence to define our scope and identify key linkages. The mhGAP (mental health Gap Action Programme) intervention guidelines first developed by WHO in 2010 and updated in 2016 with most recent evidence, provides guidelines for health providers to address mental, neurological and substance abuse disorders (MNS) in non‐specialist settings (WHO, 2010). Based on a series of reviews, the Helping Adolescents Thrive (HAT) programme developed guidelines and toolkits for the promotion of positive mental health and prevention of mental health conditions, self‐harm, substance use and other high‐risk behaviours among adolescents, ages 10–19 (WHO‐UNICEF, 2021). Disease Control Priorities (DCP3), which lays the ground for global priority MNS, includes a chapter on childhood disorders which identifies maternal mental health and parenting skills interventions as holding key potential to the reduction in prevalence of developmental and mental health conditions (Patel, 2016).

### 2.4 Conceptual framework

**Figure 1:**
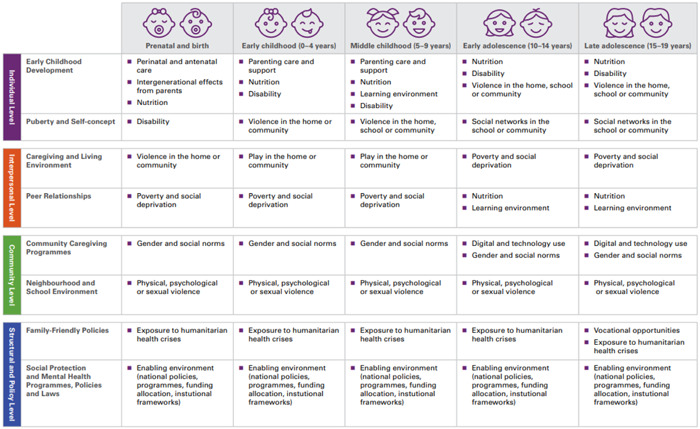
Child and adolescent mental health and psychosocial well‐being: A conceptual framework for research and evidence generation use. *Source*: Idele (2022).

The conceptual framework guiding this EGM builds upon the mental health research and evidence generation framework (see Figure 1) proposed by UNICEF Innocenti—Global Office of Research and Foresight, which was developed through a series of internal expert consultations to guide evidence and data generation efforts and to support UNICEF's Global Mental Health and Psychosocial Support Framework and programming strategy. The framework recognises that as children and adolescents grow through the life course, their interactions and influences also widen. Their mental health is thus influenced by a myriad of dynamic risk and protective factors at different layers of the social environment across their developmental stages—individual, interpersonal, community and structural and policy levels. Different factors have greater impact at different ages and across these levels.

This framework incorporates elements of other existing frameworks including: (a) the socio‐ecological model (Bronfenbrenner, 1979), which posits that a child's psychosocial well‐being depends on a myriad of factors nested within their broader social environment ranging from the household, through to the community and society levels, and the broader socio‐cultural and policy environment and can be understood as the different delivery platforms by which interventions are deployed; (b) the social determinants of health approach (Marmot & Wilkinson, 2005) which emphasises the role of circumstances in which people are born and grow up, as well as the systems in place to deal with illness; and (c) the life course epidemiology approach (Kuh, 2003) which highlights the factors and experiences over the life course and across generations that impact health outcomes at different ages and life stages.

The EGM utilises this framework by organising interventions according to delivery platforms that correspond to the levels in a child's social ecology. Further, the outcomes will be sensitive to the child's life stage and social determinants of health, thereby including child development outcomes as well. Child and adolescent mental health is complex and changes over time according to individual characteristics, relationships, context and experiences. Therefore, it is hereby understood to encompass both negative and positive mental health outcomes including well‐being and functioning as well as symptoms of distress or sadness and mental health conditions that may require specialised care.

**Figure 2:**
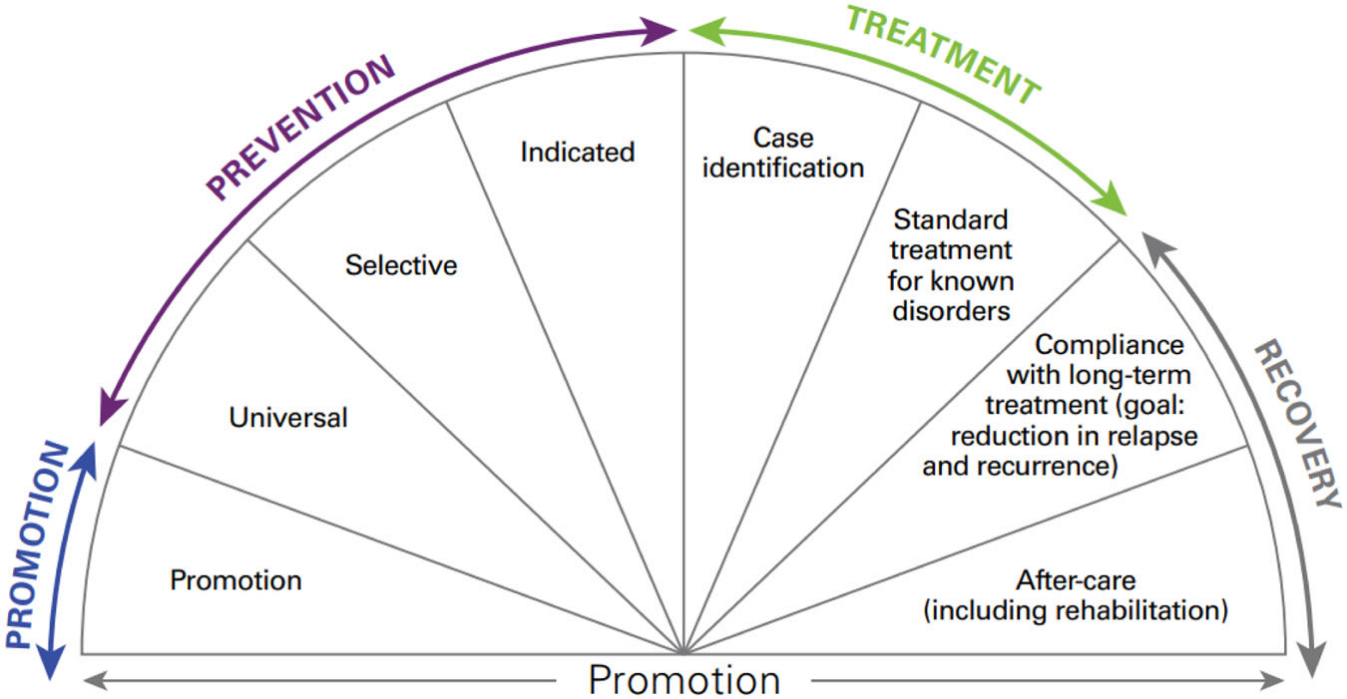
Mental health intervention spectrum. Institute of Medicine. Reducing Risks for Mental Disorders: Frontiers for Preventive Intervention Research. *Source*: Mrazek PJ, Haggerty RJ, editors. Committee on Prevention of Mental Disorders, Division of Biobehavioural Sciences and Mental Disorders. Washington, DC: National Academy Press; 1994.

Building on this, we apply the continuum of care model to categorise mental health interventions as prevention, promotion or treatment, as depicted in Figure 2 (Institute of Medicine, 1994). Mental health promotion interventions aim to enhance well‐being and create supportive and protective environments for all children and adolescents. Prevention interventions focus on preventing or reducing the risk of developing a mental health condition by targeting modifiable risk factors and can be universal (delivered to the general population, e.g., primary prevention), selective (population sub‐groups deemed to be at risk of mental health conditions developing) or indicated (populations identified at heightened risk for mental health conditions). Treatment interventions are for populations diagnosed with a mental health condition. The framework also includes recovery interventions; however, these will be excluded from the EGM.

## 3 OBJECTIVES

The objective of this EGM is to provide an overview of the existing evidence on the effectiveness of interventions aimed at promoting mental health and reducing or preventing mental health conditions among children and adolescents in LMICs.

Consistent with this, the EGM will:

1. Identify, describe and visually represent the existing evidence from systematic reviews and primary studies on the effectiveness of mental health interventions for children and adolescents.
2. Identify existing gaps in evidence to better inform practice and future primary research.
3. Identify clusters of primary studies that offer opportunities for evidence synthesis.

## 4 METHODS

### 4.1 EGM: Definitions

In contrast to systematic reviews, EGMs do not aim to synthesise the outcomes or key messages from available evidence, but instead aim to map the availability of evidence, coverage and gaps across the various dimensions of the EGM framework and make the evidence discoverable, accessible and usable. EGMs provide an overview of the existing evidence on a topic, theme or sector to signpost where evidence exists and/or where it is lacking (Bakrania, 2020). The method for this EGM is based on an a priori protocol (Sharma, 2022). We mapped evidence on the effectiveness of child and adolescent (ages 0–19) Mental health and psychosocial support (MHPSS) interventions in LMICs within the last 12 years. A description of these criteria is provided below, and an overview of search methods and sources used is presented in Supporting Information: Appendix 2.

### 4.2 Types of populations

Children and adolescents are defined as any person from 0 to 19 years of age and classified according to UNICEF's age criteria stated as follows: early childhood (0–4 years), middle childhood (5–9 years), early adolescence (10–14 years) and late adolescence (15–19 years). Primary studies where less than 50% of the sample fall within the 0–19 age range or that do not provide sufficient information of age composition will be excluded.

Population subgroups of interest includes children in alternative care, children with disabilities, LGBTQIA+ children, ethnic or racial minorities, child workers, married children, street children, children with chronic health conditions, pregnant adolescents and adolescent parents and forcibly displaced children. We also note whether studies or reviews focus on girls/females, boys/males and/or other.

LMIC are defined according to the World Bank's regional classification by country gross national income as: low‐income, lower middle‐income, upper middle‐income economies (The World Bank, 2021).

### 4.3 Types of interventions

MHPSS is defined as ‘any type of local or outside support that aims to protect or promote psychosocial well‐being and/or prevent or treat mental disorders’ (Inter‐Agency Standing Committee, 2007, p. 1). While there is no universally agreed upon categorisation of MHPSS interventions, they are hereby organised according to the levels of the social‐ecological model and UNICEF Innocenti—Global Office of Research and Foresight's conceptual framework, based on the platform of delivery:

- Individual and family‐based interventions
- School‐based interventions
- Community‐based interventions
- Digital interventions

The EGM includes filters for individual, dyad, and group‐based interventions. Under each of these categories, interventions were further categorised as mental health promotion, prevention of mental health conditions or treatment interventions. This categorisation is based on the continuum of interventions highlighted in the conceptual framework section above, and on the description for these types of interventions provided in the systematic review of MHPSS interventions for conflict‐affected children in LMICs (Jordans, 2016).

- Promotion: activities and programmes focusing on strengthening positive aspects of mental health and child well‐being
- Prevention: activities and programmes that aim to stop mental health conditions from developing, by acting on the social determinants of mental health that may be known risk factors for certain mental disorders
- Treatment: activities to reduce symptoms and improve functioning in people with identified mental disorders. We excluded studies investigating solely pharmacological treatment.

The distinction between interventions that enhance mental health and those that lower the risk of or ameliorate mental health conditions is not always clear (Supporting Information: Appendix 1; Tol, 2015). Therefore, although we include examples of interventions for each intervention type, it should be noted that there may be overlaps, for example, a cognitive behaviour therapy intervention can be classified (depending on its objectives and modules) as promotion if it is focused on building life skills or treatment if it teaches children how to cope with and overcome anxiety. Recovery interventions focused on compliance with long‐term treatment with the goal of reducing relapse and recurrence as well as after care and rehabilitation were excluded from the EGM. We excluded studies investigating the effectiveness of neurofeedback.

Table 1 lists the intervention categories and subcategories. They cover all key mental health and psychosocial support interventions across different contexts and levels of the child's social ecology, organised by platform of delivery (i.e., individual and family, school, community, and digital).

**Table 1:** Interventions.

Intervention platform	Intervention type	Examples
Individual and family‐based	Prevention	Life‐skills, maternal and paternal stimulation
	Promotion	Psychoeducation, parenting education, exercise
	Treatment	Cognitive behaviour therapy, interpersonal psychotherapy, psychological first aid
School‐based	Prevention	Peer support, life skills training
	Promotion	School based mental health promotion, peer support, life skills education
	Treatment	School counselling, group cognitive behaviour therapy
Community‐based	Prevention	Primary prevention, child friendly spaces
	Promotion	Stigma reduction, community‐based mental health advocacy
	Treatment	Task shifting and task sharing interventions, psychological first aid in the community
Digital	Prevention	Online mindfulness‐based CBT
	Promotion	Online mental health promotion, online psychoeducation
	Treatment	Computerised cognitive behaviour therapy

### 4.4 Types of outcome measures

The main outcome categories are listed in Table 2. Systematic reviews and primary studies investigating the impact of MHPSS interventions on violence prevention outcomes (addressed in the violence against children EGM; Pundir, 2020) were excluded.

**Table 2:** Outcomes.

Outcome domains	Sub‐domains
Mental health conditions	
Internalising conditions	Depression
	Anxiety disorders
	Post‐traumatic stress disorder
	Suicidal behaviour, attempt and self‐harm
	Eating disorders
Externalising conditions	Oppositional defiant disorder
	Conduct disorder
	Attention deficit hyperactivity disorder
	Alcohol and substance use
Other mental health conditions and symptoms	Other (e.g. hopelessness, anger, risk taking behaviours, cultural symptoms of distress)
Mental well‐being	
	Well‐being
	Functioning
	Ability to cope
	Social behaviour
	Social connectedness
	Other well‐being outcomes (e.g. resilience, quality of life)
Early childhood development outcomes	
	Social emotional learning
	Cognitive development
	Executive function
	Emotional regulation
	Behaviour problems

We used the International Classification of Disease (ICD‐11) criteria for including mental health conditions. The terms ‘internalising’ and ‘externalising’ refer to internally and externally focused symptoms of mental health conditions, respectively. These well‐established and widely used groupings are derived from factor analyses of psychological problems identified by clinically referred children and describe behavioural, emotional and social problems. They encompass a broad range of mental health issues and are not mutually exclusive. The mental health conditions listed under each of these are based on what emerged as the most relevant categories from our recent rapid review on the impact of child and adolescent mental health outcomes (UNICEF Office of Research—Innocenti, 2021). Further, while the medicalization and science around mental health conditions has favoured development of discrete outcomes and measures, positive mental health outcomes are yet to be standardised across the literature, with a high variability across approaches. Although positive mental health is a well‐recognised aspect of mental health, there is no established classification of positive mental health outcomes. In this EGM, we classify positive mental health outcomes according to Inter‐Agency Standing Committee Reference Group for Mental Health and Psychosocial Support in Emergency Settings' Common Monitoring and Evaluation Framework (Inter‐Agency Standing Committee, 2021). In addition, we have also included ‘other’ categories for relevant outcomes that may emerge from the included studies and relate directly to the outcomes described above (e.g., sadness or hopelessness as outcomes related to depression, or self‐efficacy and prosocial behaviours as outcomes related to mental well‐being). To capture key early childhood indicators, we looked at child development outcomes such as social‐emotional learning and cognitive development. These are critical processes through which children acquire and apply knowledge and skills to cope with challenges, manage interpersonal relationships and emotions, solve problems and make informed decisions. These indicators are also linked to later‐life mental health outcomes (Black, 2017; Patel, 2018).

### 4.5 Types of study designs

The study designs included in the EGM are systematic reviews and effectiveness studies in the form of randomised control trials and quasi‐experimental studies. We used White (2014)'s definition for quasi‐experimental studies. Mixed‐methods studies that have a randomised control trial or quasi‐experimental design with a qualitative component and a focus on intervention effectiveness will also be included. These types of study designs match the focus on intervention effectiveness.

We did not include purely qualitative studies in the EGM or quantitative and mixed‐methods studies focused on topics beyond intervention effectiveness (such as training and capacity building of practitioners and providers), studies solely on detection and diagnosis of mental health conditions, single case reports of treatments, other EGMs, systematic reviews of reviews, natural experiments and research on mental health policy or legislations.

### 4.6 Types of settings

Interventions delivered solely in hospital settings such as in‐patient and out‐patient psychiatric care are considered beyond the scope of the map's delivery platforms and were not included in the EGM.

### 4.7 Languages, publication period and types

Searches were conducted in English, but studies and reviews written in any language were be considered for inclusion. Studies in languages that could not be interpreted by any members of the review team (i.e., Farsi, Turkish, Arabic and Serbian) (*N* = 119) were excluded.

Studies published from the year 2010 onwards were included to consider the most updated and relevant search results. This date coincides with the publication of the World Health Organization's Mental Health Global Action Programme (mhGAP) intervention guidelines, providing recommendations for the treatment of mental health conditions in non‐specialist health settings (WHO, 2010). However, systematic reviews included in this EGM may include studies from before 2010.

Peer‐reviewed reports and academic papers were included. Protocols of systematic reviews and primary studies were also included and removed if the review or primary study was identified. Pilots of randomised controlled trials were excluded. Co‐registered reports were treated as duplicate reviews with data extracted from the most detailed version. Similarly, if multiple versions of the same systematic review were identified, the latest and most comprehensive version was considered for inclusion. Commentaries, conceptual or theoretical papers, editorials, conference proceedings and clinical cases were excluded.

### 4.8 Search methods and sources

#### 4.8.1 Search structure

The electronic search strategy translated concepts from the population, interventions, outcomes, and geography components of our eligibility criteria.

#### 4.8.2 Search process

A qualified information specialist (F. S.) designed and tested search strategies for each database (Sharma, 2022). All searches were conducted in December 2021 to retrieve all systematic reviews and primary studies published from January 2010 and December 2021 with no language restrictions. A draught strategy was shared with the review team and Advisory Board for comments and revisions before finalisation. The search was designed according to following Chapter 4 of the Cochrane Handbook (Higgins, 2022), peer‐reviewed using PRESS guidelines (McGowan, 2016) and reported based on PRISMA‐Search guidelines (Rethlefsen, 2021).

#### 4.8.3 Databases

A wide range of bibliographic databases, sources of grey literature, and websites were searched to cover all the relevant subject areas, geography, and study designs. The primary list of databases is as follows:

1. Systematic review repositories
  ∘ Campbell Collaboration
  ∘ 3ie Systematic Review Database
  ∘ Cochrane Library Databases
  ∘ Epistemonikos
  ∘ EPPI Centre Evaluation Database of Education Research
  ∘ Social Systems Evidence
2. Regional sources
  ∘ African Index Medicus
  ∘ African Journals Online
  ∘ Latin American and Caribbean Health Sciences Literature (LILACS)
  ∘ SciELO
3. Social science
  ∘ Applied Social Sciences Index and Abstracts (ASSIA) via ProQuest
  ∘ International Bibliography of Social Sciences (IBSS) via ProQuest
  ∘ Social Policy and Practice via Ovid SP
  ∘ Social Science Citation Index via Web of Science
  ∘ Social Science Database via ProQuest
  ∘ Social Science Research Network (SSRN)
  ∘ Sociological Abstracts (including Social Services Abstracts and Sociological Abstracts) via ProQuest
  ∘ PsycINFO via Ovid SP
4. Web and website searches
  ∘ Google Site Search (per website, including grey literature)
  ∘ UN‐affiliated relevant websites
  ∘ Subject‐focused websites
5. Health/medical databases
  ∘ CINAHL via EBSCOhost
  ∘ Cochrane Central Register of Controlled Trials (CENTRAL)
  ∘ Embase via Ovid SP
  ∘ Global Health via Ovid SP
  ∘ Global Index Medicus
  ∘ MEDLINE via Ovid SP
  ∘ PubMed (excluding MEDLINE)
6. Educational databases
  ∘ Child Development & Adolescent Studies via EBSCOhost
  ∘ Education Resources Information Center (ERIC) via ProQuest
7. Science databases
  ∘ Emerging Sources Citation Index via Web of Science
  ∘ Science Citation Index Expanded via Web of Science
  ∘ Scopus
8. Grey literature
  ∘ Google Scholar (including grey literature)
  ∘ ProQuest Dissertations and Theses Global

#### 4.8.4 Screening and study selection

Screening was conducted using EPPI‐Reviewer Web (Thomas, 2020). Given the large number of retrieved results, 5% of all titles and abstracts (*N* = 1357) were double‐screened by four reviewers (R. Y., A. I., M. S. and C. P.) and disagreements were solved by consensus. Based on this initial screening, machine learning functions within EPPI‐Reviewer (otherwise known as priority screening) were used to prioritise studies for screening. Those deemed to have a higher probability of meeting the inclusion criteria appear towards the top of the list, and the list was divided into probability groups. After revising the first 100 items within each probability group, those items within the 0%–29% probability groups were deemed of very low probability of being included and were excluded *en masse*. The remaining groups (30%–100%, *N* = 7545) were screened independently by three reviewers (R. Y., A. I. and C. P.). Next, 5% of full‐text were double‐screened by three reviewers (R.Y., A. I. and C. P.) and the remaining full‐texts were screened independently. Due to the large number of included studies and reviews, reference lists were not searched.

#### 4.8.5 Data extraction and management

Data extraction was conducted using EPPI‐Reviewer Web (Thomas, 2020). Due to the expected large volume of reviews meeting inclusion criteria, a small sample of studies and reviews were extracted by two reviewers and disagreements were resolved by consensus. The remaining coding was conducted by one reviewer independently, in consultation with other reviewers when it was necessary.

#### 4.8.6 Critical appraisal

Due to the expected large volume of studies and reviews meeting our inclusion criteria, we did not appraise the quality of included studies and reviews. Instead, we extracted data on study design and type of systematic review as well as the number of participants included in each primary study or the number of studies within each review. In this final report, we discuss the implications and sources of bias introduced by each type of design or review.

#### 4.8.7 Methods for mapping

The EPPI‐Mapper tool was used to develop the interactive online EGM.

### 4.9 Analysis and presentation

#### 4.9.1 Presentation

Each entry in the map is a systematic review or a primary study of effectiveness. The EGM identifies the number of studies covered by the map according to each intervention and outcome dimension. The available evidence is represented across two dimensions: the rows list interventions and the columns list outcome domains. Each cell shows the studies and reviews which contain evidence on that combination of intervention and outcomes or absolute gaps when no evidence exists. The number of included primary studies and reviews are represented by the size of the bubble and the type of design or review is indicated by the colour of each bubble. In addition to the dimensions (i.e., interventions and outcomes), Table 3 presents the filters of the EGM.

**Table 3:** Filters for the EGM.

Category	Data items
Context	Income level: low income; lower‐middle income; upper‐middle income; global (with at least one LMIC study included) Countries Region: East Asia & Pacific, Europe & Central Asia, Latin America & Caribbean, Middle East & North Africa, North America, South Asia, Western Central Africa, Eastern Central Africa Humanitarian context: Study or review explicitly mentions that the MHPSS intervention (as defined by IASC guidelines) was conducted in a humanitarian or conflict‐affected region. COVID‐19: Study or review explicitly mentions having been conducted in the context of the COVID‐19 pandemic or including studies conducted in the context of the COVID‐19 pandemic. Physical activity: study or review includes physical activity interventions—physical activity is ‘any body movement that is produced by the contraction of skeletal muscles that increase energy expenditure’ whereas exercise is ‘a subset of physical activity that is planned, structured and deliberate’. Format: Individual, dyad and group‐based interventions
Population	Age group: early childhood (0–4); middle childhood (5–9); early adolescence (10–14); late adolescence (15–19) Gender/sex: girl/female; boy/male; other Sub‐groups: Children in alternative care, children with disabilities, LGBTQIA+ children, ethnic or racial minorities, child labourer, married children, street children, children with chronic health conditions, pregnant adolescents and adolescent parents, and migrant and forcibly displaced children.
Study design	Systematic review Randomised controlled trial Quasi‐experimental study Mixed‐methods study Threshold of number of participants or number of studies

#### 4.9.2 Planned analysis

The EGM report provides tabulations and/or graphs of the number of studies, with accompanying narrative description, by:

- Intervention category and subcategory
- Outcome domain and subdomain
- Region and country
- Year
- Study type
- Type of intervention
- Population sub‐groups

#### 4.9.3 Stakeholder engagement

This EGM was guided by the feedback and input of an Advisory Group composed of 13 experts, researchers, practitioners and advocates from the field of global child and adolescent mental health. Age, gender, field of expertise and geographic focus have been considered when inviting members to join the Advisory Group. The group was engaged at all stages of the EGM process providing comments on the protocol, EGM online tool, in identifying ongoing primary studies and systematic reviews, reviewing the final report and providing advice on dissemination channels. Members of the Advisory Group are acknowledged in the acknowledgements.

## 5 RESULTS

### 5.1 Description of studies

#### 5.1.1 Results of the search

Upon screening completion, 697 records were included in the EGM. The EGM linked to this report can be accessed here. Supporting Information: Appendix 3 presents a flowchart of identification of studies and databases. There are many more entries in the map than there are studies. This is because the studies were coded and mapped for all the intervention categories and subcategories that they included. This also applies to the figures presented throughout this section. One intervention can be coded as more than one platform or type (promotion, prevention and treatment), based on the nature of the intervention. Similarly, one primary study or systematic review can focus on or cover more than one type of intervention platform as well. Also, more than one outcome domain and subdomain can be measured in one primary study or systematic review. Various records are counted more than once, which is necessary to provide an overview of the EGM's results.

Of these, 323 (46%) were quasi‐experimental studies, 249 (36%) were systematic reviews, 138 (20%) were randomised controlled trials and 11(2%) were mixed‐methods studies. Given our inclusion of both systematic reviews and primary studies, some primary studies are likely included within systematic reviews.

Seventy‐eight of 138 LMICs (57%) were covered in this map, including 12 Low‐income countries (44% of all low‐income countries), 31 Lower‐middle‐income countries (56% of all lower‐middle‐income countries), and 35 upper‐middle‐income countries (64% of all upper‐middle‐income countries).

Among all records of studies and reviews, we identified 24 (3%) protocols of systematic reviews and primary randomised controlled trial studies (see list of ongoing studies and reviews in ‘References’). Due to the large number of studies screened at full text, we do not provide a full list of excluded studies, but the list is available upon request.

#### 5.1.2 Description of studies

##### Interventions

Most research of mental health and psychosocial support interventions (61%, *N* = 425) conducted in LMICs, regardless of whether they focus on promotion, prevention or treatment, has been conducted in school settings (see Table 4). For instance, El‐Khodary and Samara (2020) investigated the effectiveness of a school‐based counselling programme after exposure to traumatic events among Palestinian children and adolescents in the Gaza Strip. Post‐traumatic stress disorder, anxiety, and depression symptoms of 572 students aged 12–18 were measured before the application of this treatment intervention and 2 months later.

**Table 4:** Studies and reviews by intervention platform and outcome domains.

Intervention platform	Intervention type	Mental health condition outcomes	Mental health outcomes	Early childhood development outcomes
Individual and family‐based interventions	Promotion	44	44	40
	Prevention	61	36	13
	Treatment	147	55	15
School‐based interventions	Promotion	103	129	22
	Prevention	140	65	10
	Treatment	232	91	13
Community‐based interventions	Promotion	48	62	29
	Prevention	58	32	9
	Treatment	165	81	11
Digital interventions	Promotion	19	21	4
	Prevention	31	15	0
	Treatment	39	13	3

This is followed by community‐based interventions (37%, *N* = 261), individual and family‐based interventions (34%, *N* = 237) and digital interventions (11%, *N* = 78). A total of 59% of records (*N* = 409) investigated treatment interventions, while 37% (*N* = 258) and 28% (*N* = 192) investigated promotion and prevention ones, respectively.

**Figure 3:**
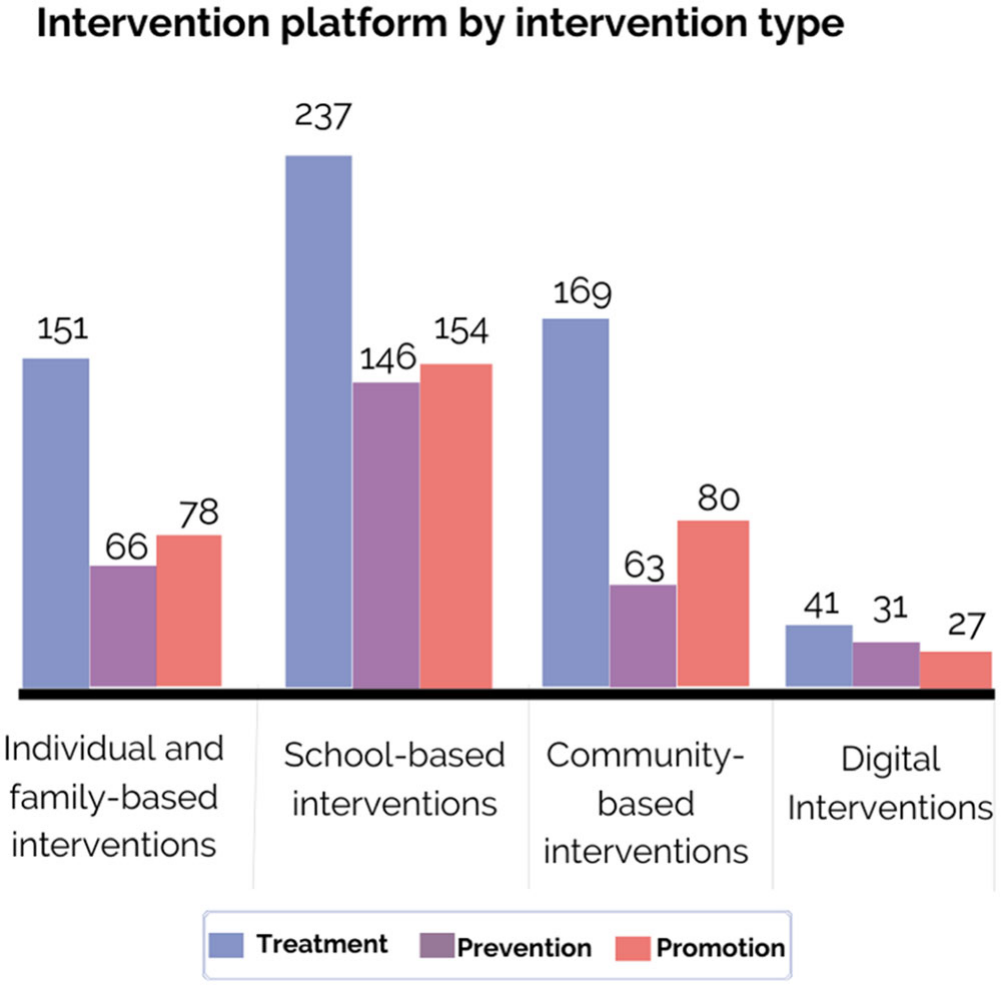
Intervention platform by intervention type.

Figure 3 shows treatment interventions as the most common researched intervention platforms. Additionally, more promotion interventions than prevention interventions are found in school‐based, community‐based, and individual and family‐based interventions. For example, based on a cluster randomised controlled trial, Zheng and colleagues (2021) assessed a peer‐to‐peer digital intervention aiming at promoting physical activity and measured whether this treatment intervention could reduce symptoms of anxiety among 954 children aged 12–14 during the COVID‐19 pandemic. However, prevention rather than promotion interventions tend to be slightly more common among digital platforms. For instance, Yap and colleagues (2020) evaluated the effectiveness of a digital game based on cognitive behavioural therapy to prevent alcohol use among 140 adolescents in the Philippines.

Most interventions covered in studies and reviews were designed for groups (79%, *N* = 553), followed by individuals (34%, *N* = 239) with just 9% directed to dyads (*N* = 62). For example, Baumgartner and colleagues (2021) conducted a cluster‐randomised controlled trial and measured the socio‐emotional early childhood development outcome after 374 mothers with their children received a group‐based integrated maternal mental health and early childhood development programme in their community in Ghana. Besides, a total of 26% of records (*N* = 184) covered interventions with more than one format. The map indicates that 24% (*N* = 166) of studies and reviews investigated both individuals and groups, compared to 5% (*N* = 39) investigated both dyads and groups and 4% (*N* = 31) investigated both individual and dyads. For instance, Anttila and colleagues (2019) investigated the effectiveness of a digital web programme, which was conducted in both small groups and individually, on the well‐being of 180 Thai adolescents. Four percent of records (*N* = 26), which are all systematic reviews, researched all three formats. Eight percent of studies and reviews (*N* = 53) did not report the format of provision.

A total of 82 studies and reviews included interventions consisting of physical activities. Most of them were designed in school‐based settings (*N* = 66) and for treatment (*N* = 51). For example, Cocca and colleagues (2020) measured psychological well‐being, self‐esteem, stress, and anxiety among 252 Mexican schoolchildren ages 10–12 before and after they attended a 6‐month game‐based physical education programme at school. This type of interventions was more frequently delivered to early (*N* = 67) and late adolescents (*N* = 55) than early (*N* = 11) and middle childhood (*N* = 45) and most were delivered in groups (*N* = 69).

##### Outcomes

**Figure 4:**
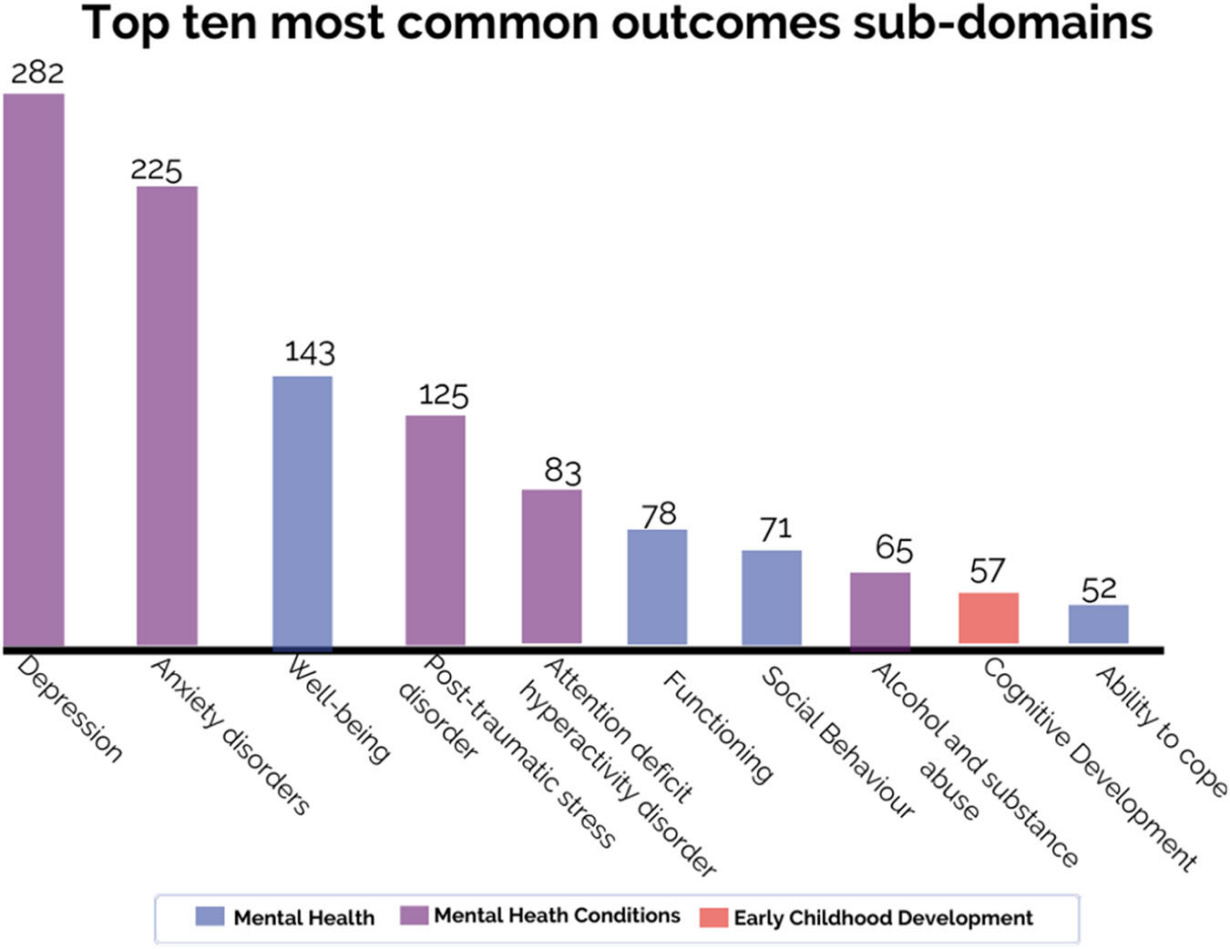
Top 10 most common outcome sub‐domains. This figure only shows the top 10 most common sub‐domains which represent 83% of all coded outcomes and excludes the other nine less common sub‐domains.

Most studies investigated mental health conditions (83%, *N* = 578), followed by mental health (46%, *N* = 320) and early childhood development outcomes (11%, *N* = 76). Figure 4 presents the top 10 most common outcome sub‐domains.

As presented in Table 5, depression (40%, *N* = 282) was the most frequently analysed outcome sub‐domain, followed by anxiety disorders (32%, *N* = 225), well‐being (21%, *N* = 143), and post‐traumatic stress disorder (18%, *N* = 125). It is also notable that ‘other mental health problems and symptoms’ and ‘other mental well‐being outcomes’ are among the most frequently measured sub‐domains. For example, in a randomised control trial, Barron and colleagues (2016) measured the effectiveness of teaching recovery techniques on dissociation, war stressors, posttraumatic stress and depression, among 154 Palestine adolescents.

**Table 5:** Studies and reviews by outcome domains and sub‐domains.

Outcome domains	Sub‐domains	Number of studies and reviews	Frequency (%)
Mental health conditions	Depression	282	40
	Other mental health problems and symptoms	264	38
	Anxiety disorders	225	32
	Post‐traumatic stress disorder	125	18
	Attention deficit hyperactivity disorder	83	12
	Alcohol and substance abuse	65	9
	Conduct disorder	32	5
	Suicidal behaviour, attempt and self‐harm	31	4
	Oppositional defiant disorder	27	4
	Eating disorders	15	2
Mental health	Other mental well‐being outcomes	201	29
	Well‐being	143	21
	Functioning	78	11
	Social behaviour	71	10
	Ability to cope	52	7
	Social connectedness	48	7
Early childhood development outcomes	Cognitive development	57	8
	Behaviour problems	30	4
	Social‐emotional learning	20	3
	Executive function	20	3
	Emotional regulation	19	3

All five sub‐domains in early childhood development outcomes were covered by less than 10% of studies and reviews. Within mental health outcomes, two of six sub‐domains were measured by less than 10% of studies and reviews, which were ability to cope or social connectedness. Five sub‐domains in mental health conditions outcomes—eating disorders, oppositional defiant disorder, suicidal and self‐harm behaviour, conduct disorder, and alcohol and substance abuse are also covered by less than 10% of studies and reviews.

The most measured combinations of sub‐domains were depression and anxiety disorders (24%, *N* = 165), and depression and post‐traumatic stress disorder (12%, *N* = 85). Post‐traumatic stress disorder, depression and anxiety disorders are another common combination of sub‐domains, included in 50 (7%) studies and reviews.

**Figure 5:**
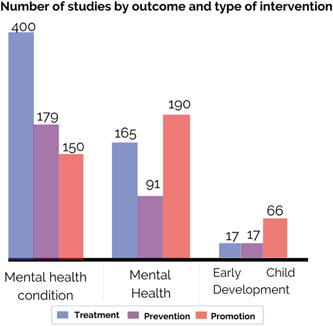
Number of studies by outcome and type of intervention.

Figure 5 shows the number of results by outcome groups and intervention type. Most studies of interventions addressing mental health conditions, whether they focus on internalising, externalising, or other mental health problems, most studies focus on treatment. For instance, Fatemi Nayeri and colleagues (2021) investigated the efficacy of group reality therapy in attention deficit hyperactivity disorder and oppositional defiant disorder among 40 Iranian adolescents ages 12–18.

##### Age groups

**Figure 6:**
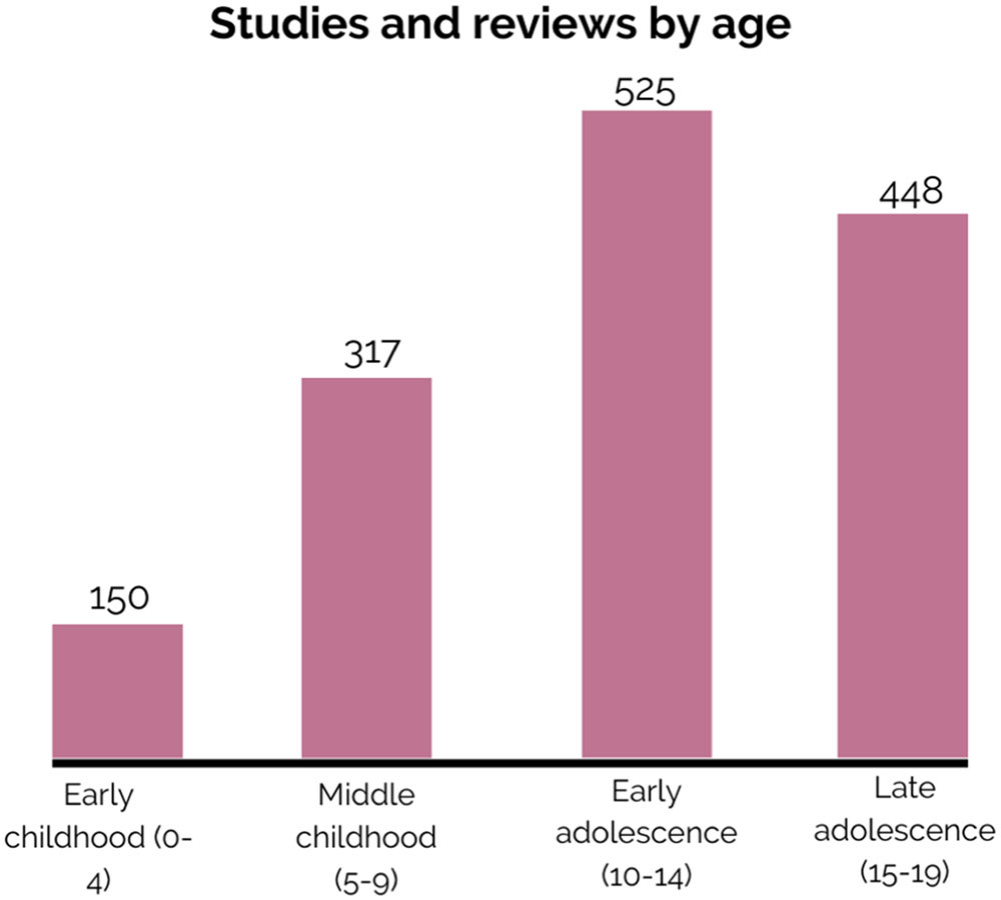
Distribution of studies and reviews by age.

**Figure 7:**
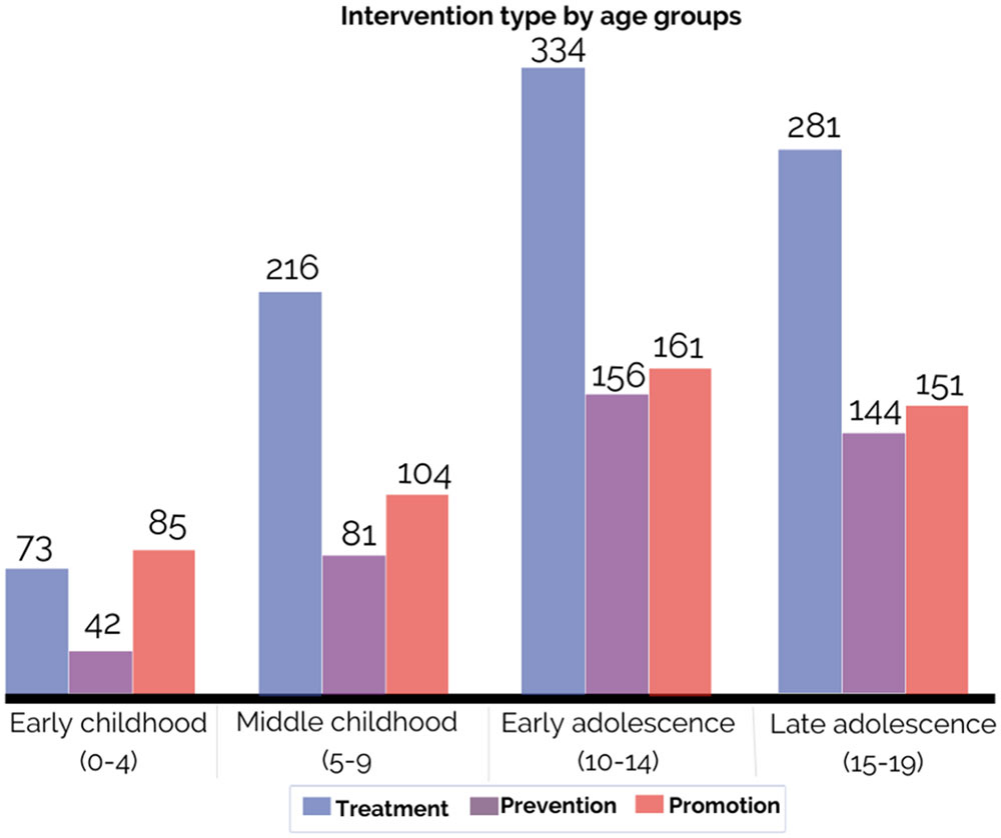
Distribution of studies and reviews by intervention type and age.

Most included studies and reviews investigated the effectiveness of mental health and psychosocial support interventions in early (75%, *N* = 525) and late adolescence (64%, *N* = 448), followed by 45% (*N* = 317) of studies and reviews which investigated middle childhood, and 22% (*N* = 150) on early childhood (Figure 6). Most studies looked into treatment interventions across all age groups with the exception of early childhood, for which similar number of studies investigated treatment (10%, *N* = 73) and promotion (12%, *N* = 85) interventions, respectively (Figure 7).

##### Populations

**Figure 8:**
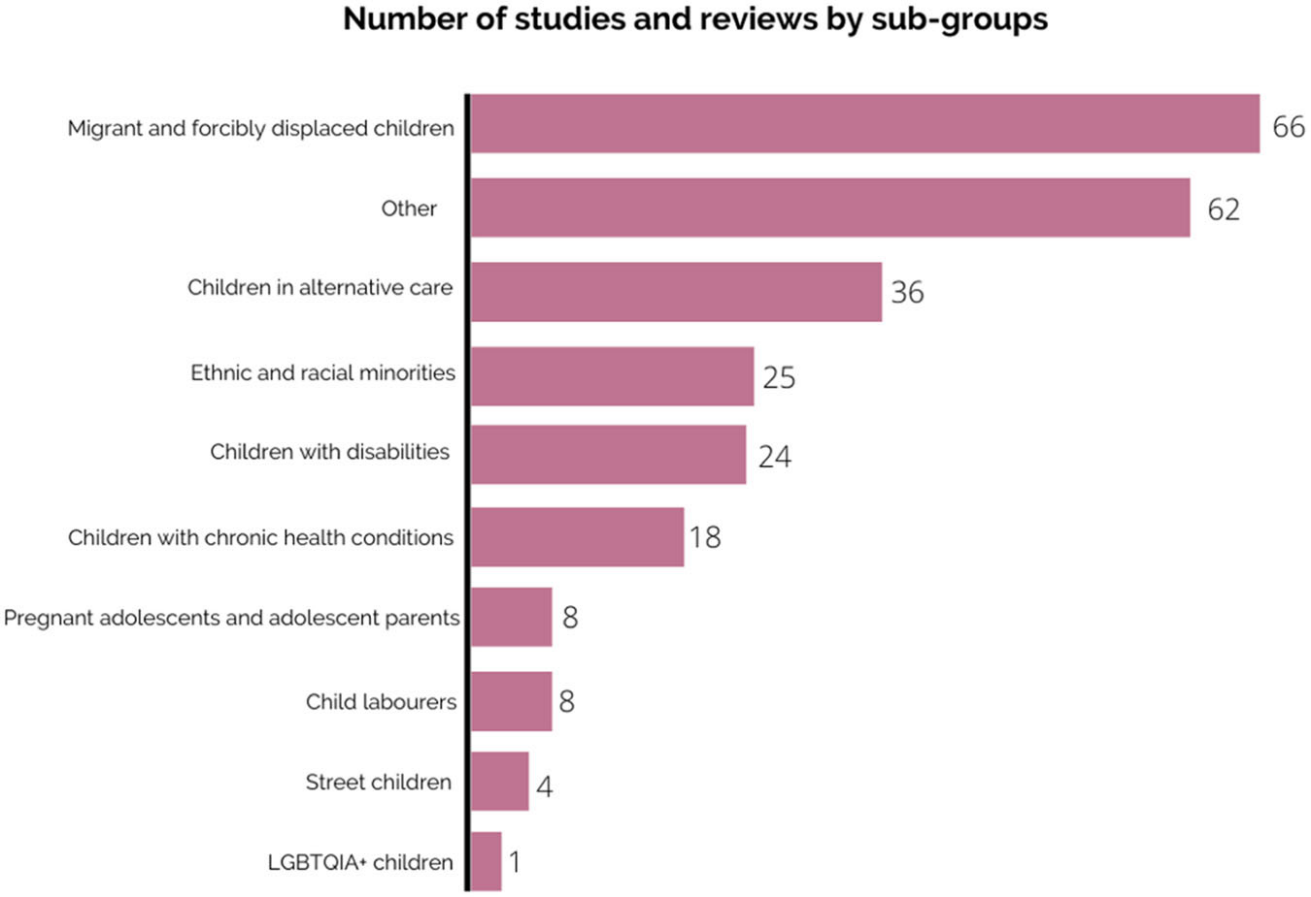
Number of studies and reviews by sub‐groups.

As presented in Figure 8, less than one‐third of studies and reviews (31%, *N* = 213) covered or focused on specific populations. Most explicitly included or targeted sub‐groups were migrants and forcibly displaced children (10%, *N* = 66), children in alternative care (5%, *N* = 36), ethnic and racial minorities (4%, *N* = 25), children with disabilities (4%, *N* = 24). No studies focused on child marriage and only one focused on non‐binary gender identities (Damanpak‐Rizi, 2021). Using a randomised control study design, Damanpak‐Rizi and colleagues (2021) investigated the effectiveness of a cognitive‐behavioural online family‐based intervention on family violence against transgender youth in Iran and analysed depression, anxiety, suicide thoughts and attempts and self‐esteem among 50 participants as secondary outcomes. A few studies and reviews focused on pregnant adolescents/adolescent parents, children living in the streets and child labourers. For instance, Watters and colleagues (2016) synthesised evidence on MHPSS interventions for children and adolescents in street situations in LMICs from five studies.

There were 62 studies and reviews investigating other sub‐groups such as children of parents with severe mental illness, victims of sexual abuse and other forms of violence and youth offenders. For example, Wannachaiyakul and colleagues (2017) investigated the effectiveness of the computerised cognitive behavioural therapy programme for reducing depression among 84 adolescents with delinquency problems in Thailand, while Robjant and colleagues (2019) researched the effectiveness of an adapted narrative exposure therapy treatment on post‐traumatic stress symptoms and aggression among 92 former female child soldiers in Democratic Republic of the Congo.

Most studies focused on both girls/female and boys/male (84%, *N* = 586). There are 55 studies and reviews (8%) that only focused on girls, while 37 studies and reviews (5%) only focused on boys.

**Figure 9:**
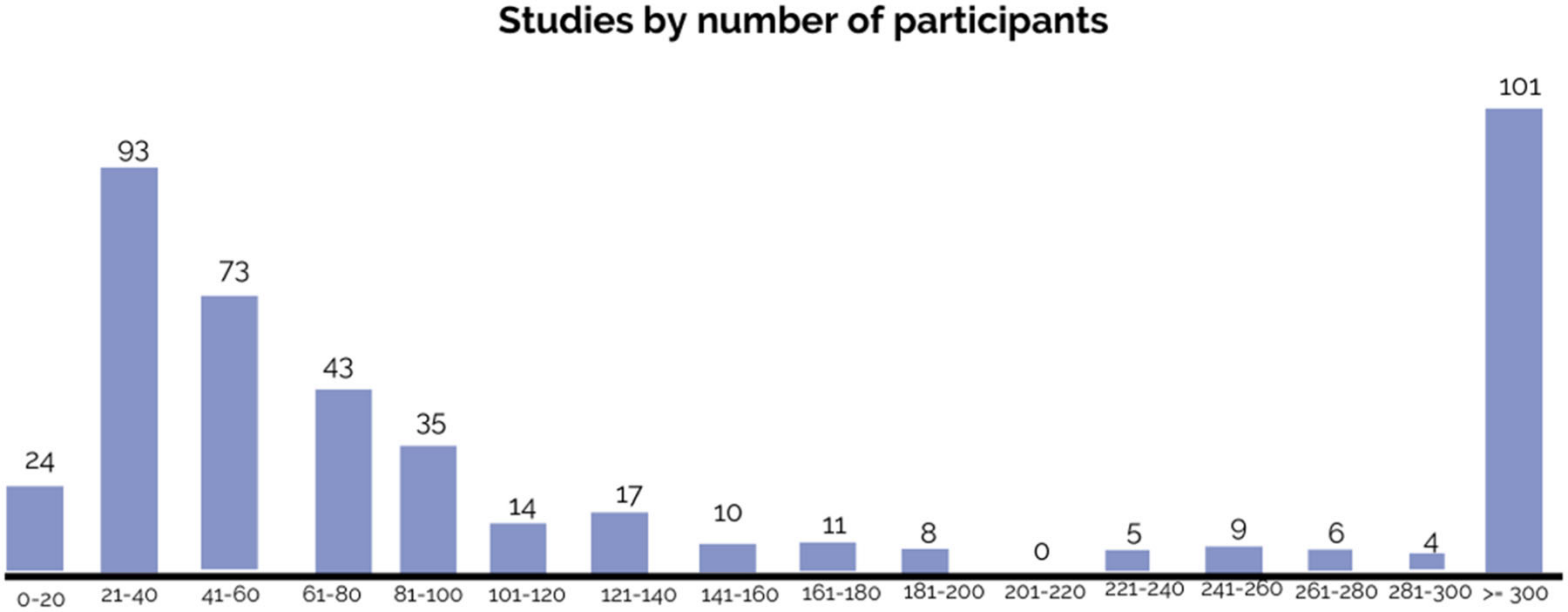
Number of studies by sample size.

Among all primary studies, about two‐third are quasi‐experimental studies (67%, *N* = 307), followed by 31% randomised controlled trials (*N* = 140), and 2% mixed‐methods studies (*N* = 11). As presented in Figure 9, 5% of primary studies (*N* = 24) consist of a sample size of fewer than 20 participants, and 41% of primary studies (*N* = 190) has equal to or fewer than 60 participants. This finding suggests a large number of studies rely on small sample sizes which might not accurately reflect the characteristics of the larger group.

About 22% of primary studies (*N* = 101) have more than 300 participants. Most of these primary studies (86%, *N* = 87) focus on group‐based interventions, compared to 13 looking at individual‐based ones and 10 dyad ones. By intervention platform, nearly 60% of these primary studies (59%, *N* = 60) focus on school‐based interventions, compared to 30 community‐based interventions, 22 individual and family‐based interventions, and 4 digital interventions. There are 58 studies (57%) focusing on group‐based interventions in school settings with a sample size larger than 300.

**Figure 10:**
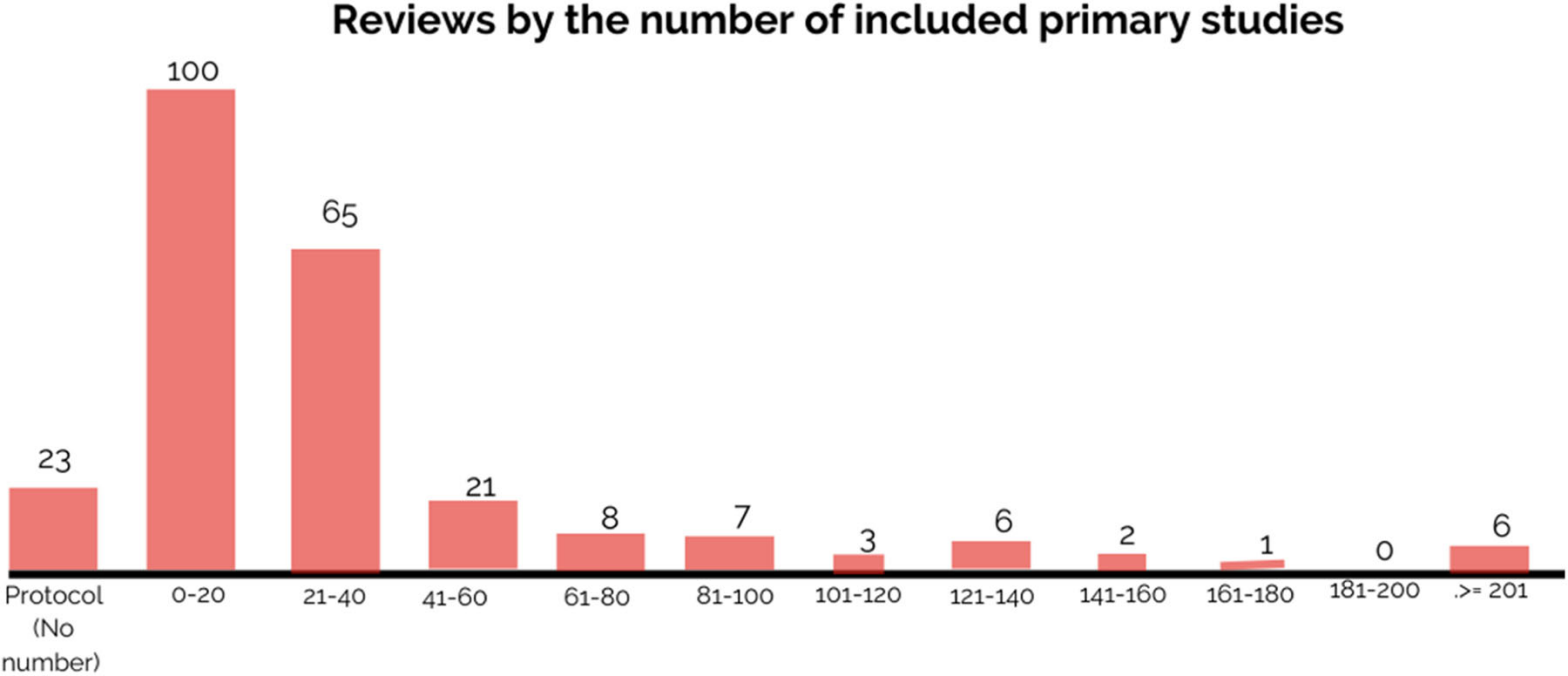
Number of reviews by included primary studies.

As presented in Figure 10, among all reviews except protocols (*N* = 216), 46% of reviews (*N* = 100) contain equal or fewer than 20 primary studies. Only less than one quarter of reviews (24%, *N* = 51) have equal to or more than 40 primary studies.

##### Settings

This map covered 78 of 138 LMICs (57%), including 12 low‐income countries (44% of all low‐income countries), 31 lower‐middle‐income countries (56% of all lower‐middle‐income countries) and 35 upper‐middle‐income countries (64% of all upper‐middle‐income countries). Various studies (mostly systematic reviews) covered both high‐income countries and LMICs and were classified as global (31%, *N* = 219). We coded the country of a study or review according to the country in which the research was conducted. A study or review may be coded for more than one country.

**Figure 11:**
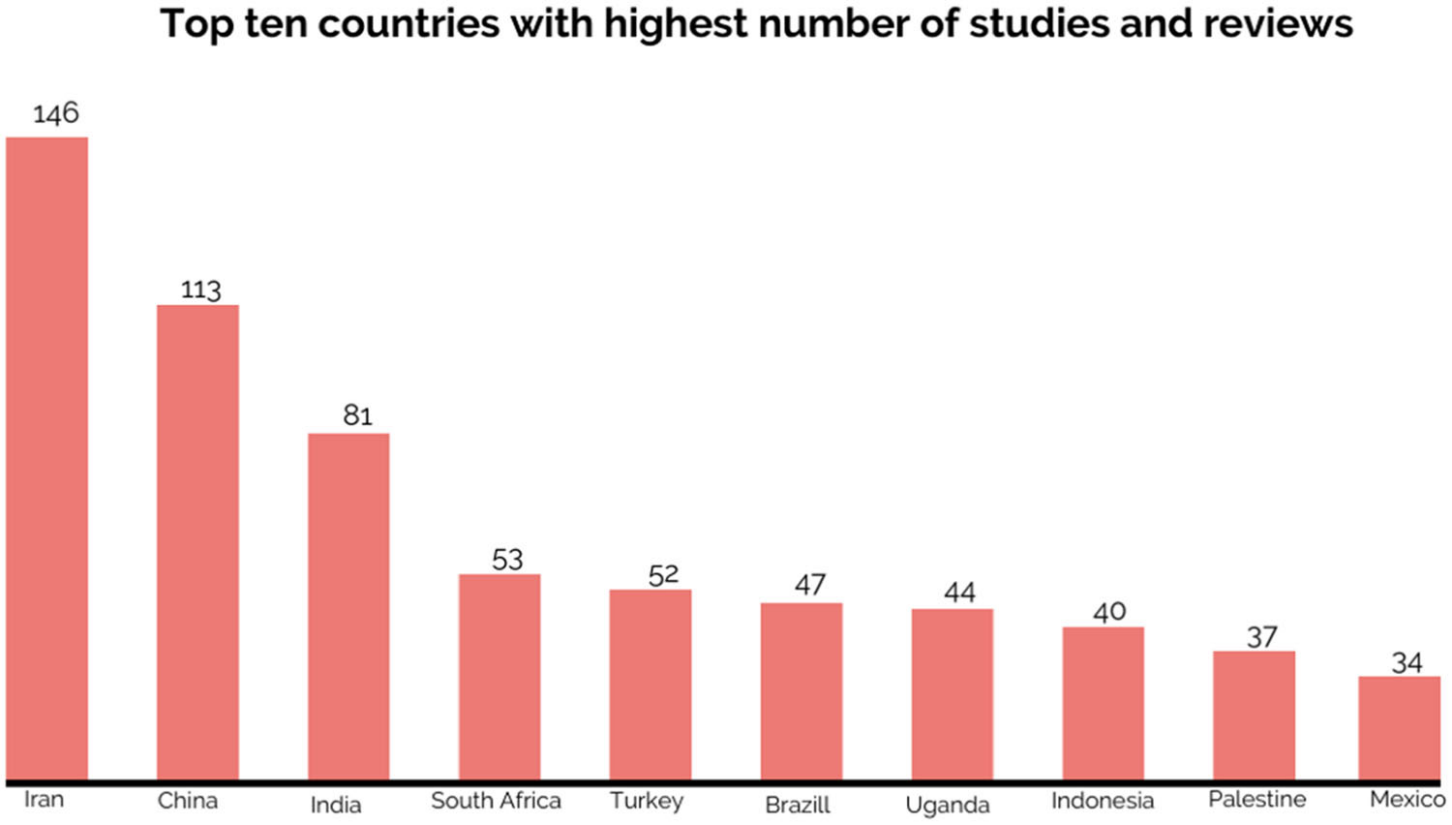
Top 10 most researched countries.

The top 10 LMICs that were covered by most studies and reviews were presented in Figure 11. Most studies and reviews were conducted in Iran (21%, *N* = 146), followed by China (16%, *N* = 113) and India (12%, *N* = 81).

**Figure 12:**
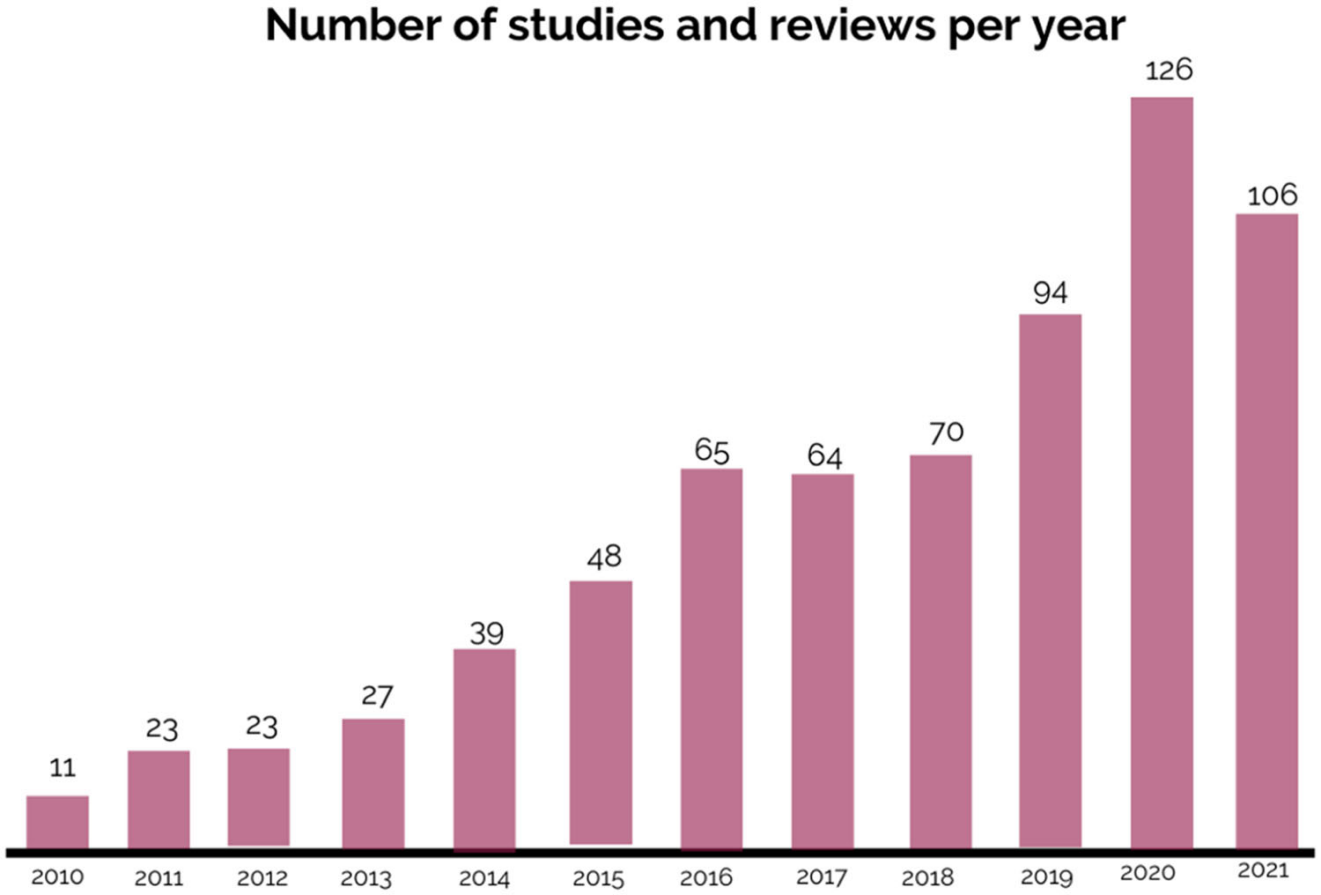
Number of publications per year.

With an average of 58 studies and reviews published yearly, showcasing data from LMICs, Figure 12 indicates the field of child and adolescent mental health has been expanding progressively during the last 12 years with a 16% average year‐on‐year increase rate, and a 41% compound annual growth rate in the number of publications.

**Figure 13:**
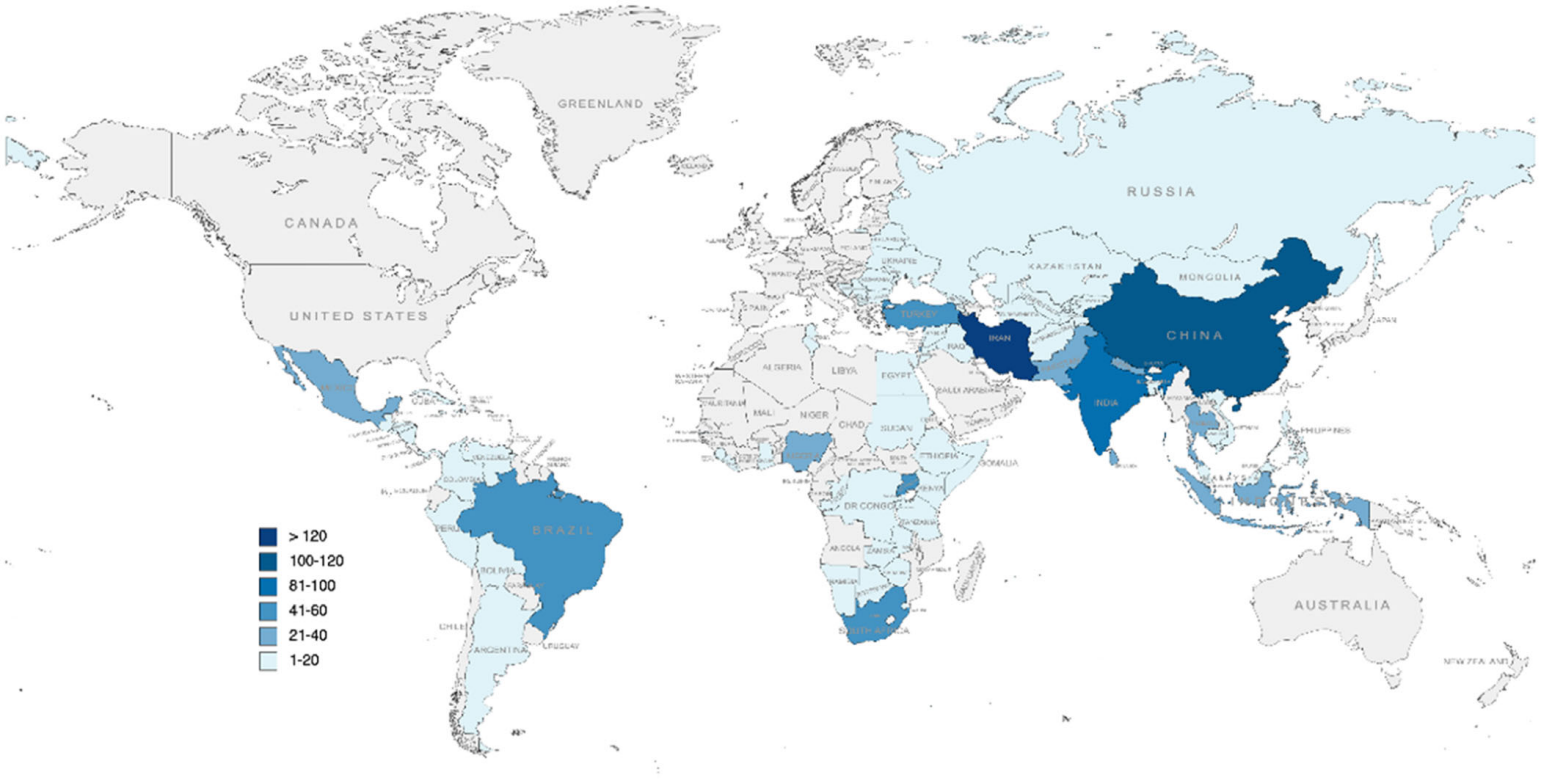
Geographic distribution of studies. All HICs are not labelled in any colour in this map as they are out of the scope. Any LMIC in grey colour means no study or review were conducted in this country. The designations employed in this publication and the presentation of the material do not imply on the part of UNICEF the expression of any opinion whatsoever concerning the legal status of any country or territory, or of its authorities or the delimitations of its frontiers.

Figure 13 indicates the geographic distribution of identified studies and studies within reviews.

**Figure 14:**
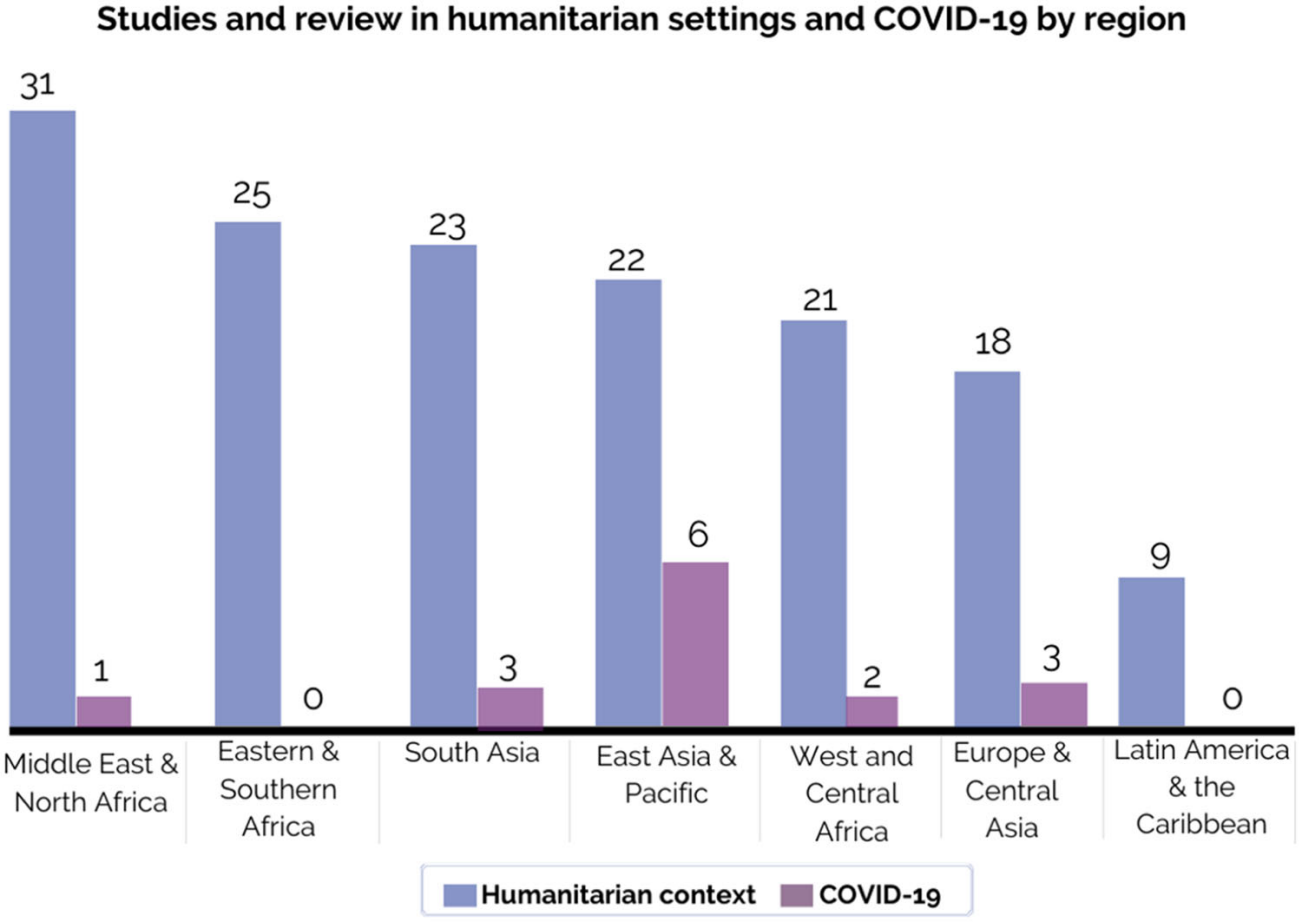
Studies and reviews in humanitarian settings and COVID‐19 by region.

A total of 67 (10%) studies were conducted in humanitarian settings (e.g., natural disaster, armed conflict, complex political emergencies) and we identified 9 (1%) studies with a focus on interventions aimed at tackling the impact of COVID‐19 on the mental health of children and adolescents in LMICs. For example, Dhital and colleagues (2019) examined whether a psychosocial support training programme for schoolteachers could improve mental health and hope among 1220 adolescents in an earthquake‐affected area in Nepal. As presented in Figure 14, studies in humanitarian settings were covered in all regions, while there were no studies including COVID‐19 conducted in Eastern and Southern Africa or Latin America and Caribbean. The Middle East and North Africa region has the most studies in humanitarian settings, but only one in COVID‐19. East Asia and Pacific have the most studies that researched the effectiveness of MHPSS interventions during the pandemic. For instance, Xu and colleagues (2021) explored the effect of the aerobic exercise intervention in combination with acceptance and commitment therapy on mental health among 90 adolescents ages 12–19 in China during the outbreak of COVID‐19 pandemic.

###### Region

Most studies were conducted in the Middle East and North Africa (33%, *N* = 228), although this is mainly driven by the large number of studies and reviews focused on Iran. East Asia and Pacific (31%, *N* = 217) is the second most represented region, followed by Europe and Central Asia (28%, *N* = 198), South Asia (20%, *N* = 139), Latin America and the Caribbean (19%, *N* = 135) and Eastern and Southern Africa (19%, *N* = 134). Only 9% of research (*N* = 63) identified through this EGM was conducted or focused on West and Central Africa.

The map covered 78 LMICs while we did not identify any studies conducted in 60 LMICs within the last 12 years.

####### Middle East and North Africa

The EGM includes studies from 10 of 13 LMICs in this region. No studies were identified in Algeria, Djibouti, or Morocco. More than half of the studies and reviews in this region included interventions delivered at schools (*N* = 148) and designed for treatment (*N* = 155). School‐based treatment interventions alone were included in 102 studies and reviews. Most interventions were delivered in groups (*N* = 185). In contrast, digital interventions were the least covered platform (*N* = 23), and prevention interventions were the least covered type of intervention (*N* = 54).

Most studies and reviews measured internalising problems (*N* = 140), within which post‐traumatic stress disorder, depression, and anxiety disorders were the most researched conditions and suicidal behaviour and attempt (*N* = 7) and eating disorders (*N* = 1) the least investigated ones. Except for attention deficit hyperactivity disorder and well‐being, all the rest of the sub‐domain outcomes were much less covered. Adolescents were more likely to be in the study population than in early and middle childhood. Most studies in this region consisted of a sample of fewer than 60 participants. In the Middle East and North Africa, 39 studies and reviews focused on migrant and forcibly displaced children in this region, which is the most among all regions. The only study that covered LGBTQIA+ children on our map was conducted in this region, while pregnant adolescents/adolescent parents, and street children were also researched, although only in one study/review.

####### East Asia and Pacific

Only 8 of 23 LMICs in the region were covered in this map. American Samoa, Fiji, Kiribati, DPRK, Lao PDR, Marshall Islands, Micronesia, Myanmar, Samoa, Solomon Islands, Timor‐Leste, Papua New Guinea, Tonga, Tuvalu, or Vanuatu were not identified in any studies or reviews.

About half of the studies and reviews focused on this region investigated interventions designed for treatment (*N* = 124) and promotion (*N* = 92). School‐based interventions (*N* = 153) were the most popular intervention platforms, while both individual and family‐based interventions (*N* = 89) and community‐based interventions (*N* = 97) were frequently studied, in comparison to digital interventions (*N* = 36). Most interventions were delivered in groups (*N* = 185). School‐based treatment interventions alone were included in 86 studies and reviews. Depression (*N* = 118), anxiety disorders (*N* = 88), and well‐being (*N* = 52) were the three most measured outcomes. Half of the studies and reviews in this region focused on school and community‐based interventions for depression (*N* = 103). Most studies and reviews focused on late (*N* = 175) and early adolescence (*N* = 169). No studies covered street children, married children, or LGBTQIA+ children in this region. Child labourer and pregnant adolescents/adolescent parents were only covered in two reviews.

####### Europe and Central Asia

This map covered 18 of 22 LMICs in this region, leaving Albania, Andorra, Azerbaijan, and Montenegro uncovered. More than half of the studies and reviews in this region investigated interventions that were designed for treatment (*N* = 128) and were conducted in school settings (*N* = 133). Individual and family‐based interventions (*N* = 96) and community‐based interventions (*N* = 106) were also frequently studied. Treatment interventions in individual and family settings and community settings were equally commonly researched. Post‐traumatic stress disorder, depression, anxiety disorders, and well‐being were the most frequently measured sub‐domains. Most studies and reviews focused on early adolescents (*N* = 174). Most interventions were implemented in groups (*N* = 162). No studies covered street children, married children, or LGBTQIA+ children in this region.

####### South Asia

In this region, six out of eight LMICs were covered, except Bhutan and Maldives. More than half of the studies and reviews in this region investigated interventions that were designed for treatment (*N* = 84), and school‐based (*N* = 100). Treatment interventions in school settings were included in 64 studies and reviews. Post‐traumatic stress disorder, depression, and anxiety disorders were the three most measured sub‐domain outcomes. Most interventions were provided in groups (*N* = 115). Most studies and reviews focused on early adolescents (*N* = 113). No studies covered married children or LGBTQIA+ children in this region.

####### Latin America and the Caribbean

In this region, 16 of 26 LMICs were covered. The following LMICs were not identified in any studies or reviews: Belize, Costa Rica, Dominica, Dominican Republic, Ecuador, Grenada, Guyana, Paraguay, Saint Vincent and the Grenadines, and Suriname.

More than half of the studies and reviews in this region investigated interventions that were designed for prevention (*N* = 72) and were provided at schools (*N* = 93). School‐based prevention interventions were included in 61 studies and reviews. Most interventions were delivered in groups (*N* = 112). Depression, anxiety disorders, and alcohol and substance use disorders were the three most measured sub‐domain outcomes. Interventions for alcohol and substance use disorders were commonly conducted in school settings, and most of them were prevention interventions. More studies and reviews focused on early adolescents (*N* = 107). No studies covered married children or LGBTQIA+ children in this region.

####### Eastern and Southern Africa

In the region, 14 of 22 LMICs were covered. No studies or reviews were conducted in Angola, Comoros, Eritrea, Lesotho, Madagascar, Malawi, Mozambique, or South Sudan. More than half of the studies and reviews in this region investigated interventions that were school‐ (*N* = 82), and community‐based (*N* = 80), and designed for treatment (*N* = 86). Community‐based treatment interventions were covered by 53 studies and reviews and 52 studies covered school‐based treatment interventions. Most interventions were provided in groups (*N* = 113). Depression, post‐traumatic stress disorder, and anxiety disorders were the three most measured sub‐domain outcomes. More studies and reviews focused on early (*N* = 115) and late adolescents (*N* = 102). No studies covered married children or LGBTQIA+ children in this region.

####### West and Central Africa

Only 6 of 24 LMICs were covered in this region. The following countries were not identified in any studies or reviews: Benin, Burkina Faso, Cabo Verde, Cameroon, Central African Republic, Chad, Côte d'Ivoire, Equatorial Guinea, Gabon, Guinea, Guinea‐Bissau, Liberia, Mali, Mauritania, Niger, Sao Tome and Principe, Senegal and Togo.

More than half of the studies and reviews in this region investigated interventions that were school‐ (*N* = 44), and community‐based (*N* = 43), and designed for treatment (*N* = 49). Most interventions were provided in groups (*N* = 57). Depression, post‐traumatic stress disorder, and anxiety disorders were the three most measured sub‐domain outcomes. No studies or reviews measured eating disorders or oppositional defiant disorder. More studies and reviews focused on early (*N* = 56) and late adolescence (*N* = 54). No studies covered married children or LGBTQIA+ children in this region.

##### Study designs

**Figure 15:**
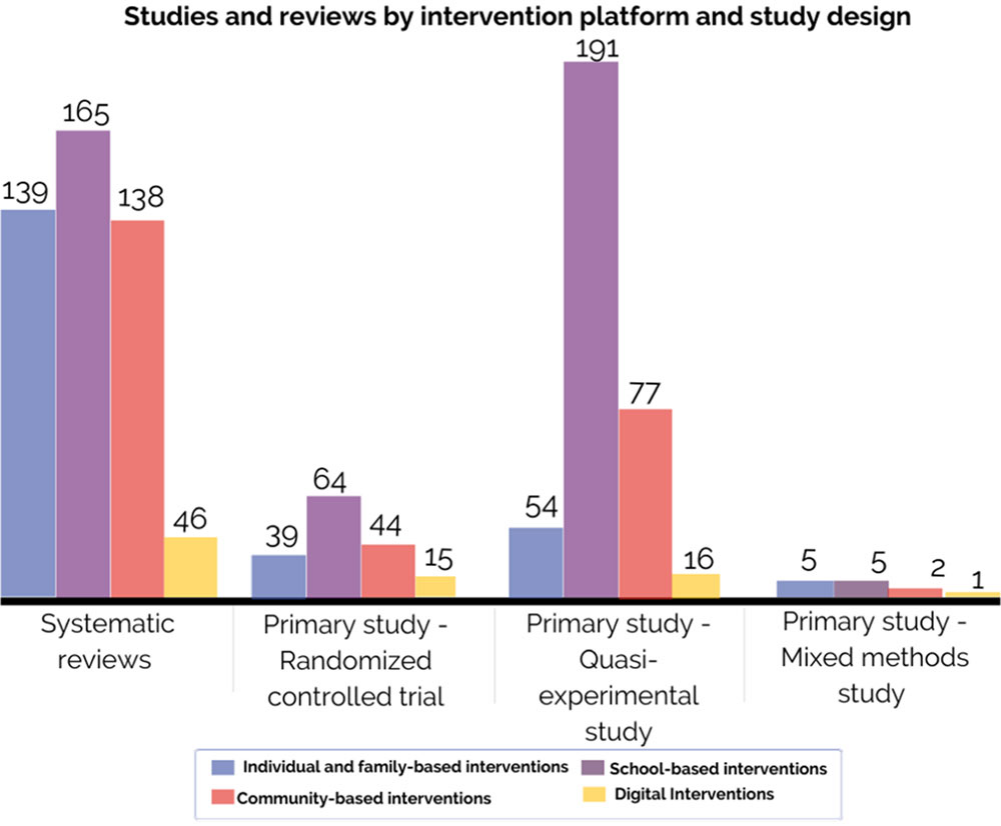
Studies and reviews by intervention platform and study design.

Most records were quasi‐experimental studies (44%, *N* = 307), followed by systematic reviews including protocols (34%, *N* = 239) and randomised controlled trials including protocols (20%, *N* = 140). As presented in Figure 15, school‐based interventions are the most common intervention platform covered across these three publication types, followed by community‐based intervention as the second and individual and family‐based interventions as the third.

## 6 DISCUSSION

Child and adolescent mental health in low‐resource settings is inadequately understood and developed as a field (Kumar, 2021). A lack of understanding of the available evidence to improve child and adolescent mental health in these settings represents a key obstacle to its development. The challenge is thus to identify promising interventions and the most urgent research gaps within this neglected field. In this EGM, we feature evidence from 697 primary studies and systematic reviews conducted within the last 12 years on child and adolescent mental health interventions in LMICs. While we are not able to synthesise the findings from this large pool of studies, we provide an overview of the available evidence, gaps and recommendations.

### 6.1 Areas of evidence concentration and gaps

#### 6.1.1 Treatment is prioritised over promotion and prevention interventions

A principal finding of this EGM is that most of the evidence on child and adolescent mental health is reactive rather than proactive, with a strong focus on the treatment of mental health conditions over prevention or promotion interventions. This finding is consistent across all regions except for Latin America and the Caribbean, where prevention interventions make up the largest pool of researched interventions and across all age groups, with the exception of studies and reviews on early childhood, for which a similar number of studies investigated treatment and promotion interventions. While this suggests progress in terms of the availability of knowledge of which treatment intervention to implement (or not), evidence is needed on interventions aimed at strengthening positive aspects of child and adolescent mental health and psychological wellbeing, empower children and adolescents to live healthy lives, respond to early signs of distress, foster pro‐social behaviours, resilience, self‐esteem and coping mechanisms. Research is also needed on prevention approaches, including universal, selective and indicative, aimed at reducing the likelihood of developing future mental health conditions. Childhood and adolescents are crucial periods for healthy developments and during this period, interventions aimed at equipping them with necessary skills, supports and resources play a fundamental role in preventing the development of mental health and psychosocial problems later in life. Current evidence is likely insufficient to determine which promotion and prevention interventions provide the best results for child and adolescent mental health in these settings. This finding also reveals a disconnect with practice as most implemented MHPSS interventions are psychosocial interventions aiming to strengthen community and family support and promote mental health which and are not regularly provided by clinicians.

Evidence of the large and persistent gap between the number of people that *need* mental health care and the number of people who *receive* it, has driven an increase recognition on the importance of mental health globally. However, the solution to this global treatment gap is not solely additional access to treatment interventions. Given the magnitude of the global burden of mental health conditions, treatment alone will not be enough to close this gap (Purgato, 2020). In pursuit of reducing the burden of mental health conditions, strategic priority and global mental health research funding need to be directed to research on promotion and prevention interventions that can be feasibly delivered in LMICs. More evidence on these types of interventions is required, especially in crucial developmental stages such as early childhood.

#### 6.1.2 Schools are the most researched platform for mental health interventions

Schools are one of the most important community settings for the provision of mental health interventions. In this EGM, schools emerged as the most researched platform for mental health action. Regardless of the type of intervention (i.e., prevention, promotion, or treatment), most mental health research is conducted at educational settings and focuses on early and late adolescence. Schools are one of the most convenient locations (i.e., feasibility and cost‐effectiveness) for reaching a wide number of children and adolescents and their families (Barry, 2013). Evidence from systematic reviews suggests that mental health programmes incorporating life skills, social and emotional learning to address emotional and behavioural problems can improve for children's emotional and social functioning, including reduced depression and anxiety and improved coping skills (Barry, 2013). Although future systematic reviews are needed, considering that nearly 100% of published studies in psychology confirm their initial hypothesis (Haeffel, 2022), there is likely evidence supporting the use of MHPSS interventions in school settings. Despite the potential for early intervention in educational settings, a recent review of the effectiveness of universal school‐based mental health interventions highlights the lack of evidence supporting the use of preventive interventions (Bradshaw, 2021). Gaps remain on the effectiveness of mental health and psychosocial support interventions during early childhood and for children without school access.

#### 6.1.3 Limited evidence from West and Central Africa

Children and adolescents ages 10–19 constitute 23% of the population in West and Central Africa, making it one of the youngest regions in the world (UNICEF, 2019). Children and adolescents in this region are also disproportionally affected by key risk factors for mental health conditions. In these settings, the epidemiology of mental health is determined by a wide range of socio‐economic factors, including structural inequality, displacement, armed conflict, poor or no implementation of health and social policies and scarce human and financial resources for mental health care. Only 9% of research identified through this EGM was conducted or focused on West and Central Africa. Lack of data on effective interventions hamper efforts to improve the mental health of children and adolescents in the region.

#### 6.1.4 Moving beyond discrete categorisation of outcomes

A large subset of studies and reviews focus on interventions addressing internalising problems, such as depression, anxiety disorders, and post‐traumatic stress disorder and these conditions are frequently investigated together. The increased prevalence of these conditions and high comorbidity means that they are often researched simultaneously and conceptualised as ‘common mental health conditions’. Although depression was the most frequently researched mental health outcome, it is followed by ‘other mental health conditions’ (e.g., hopelessness, anger, risk‐taking, aggressive behaviours) and ‘other mental health outcomes’ (e.g., resilience, quality of life). This could indicate a move towards more nuanced, contextually and culturally relevant manifestations of mental health problems or well‐being.

#### 6.1.5 Limited evidence of humanitarian settings

Only 10% of studies and reviews focused on investigating the effectiveness of MHPSS interventions for children and adolescents living in humanitarian settings. Prolonged exposure to conflict, mass displacement, violence and natural disasters put children and adolescents at greater risk of developing mental health and psychosocial problems, threaten children's ability to grow healthily and often prevent parents and caregivers from provided care, protection and support. Meeting these challenges requires building an evidence base of effective MHPSS interventions that consider the different contextual factors of these settings as well as the implementation challenges, which are repeatedly neglected.

#### 6.1.6 Limited evidence on digital interventions

Although the use of digital technologies to educate, facilitate diagnosis and support treatment have been proposed as a future direction for global mental health (Patel, 2018), we identified very limited evidence on the effectiveness of digital interventions across all outcomes. Despite the known potential of digital interventions to overcome barriers such as stigma, geographical and time limitations, this field remains understudied. The COVID‐19 pandemic also produced new opportunities for web‐based and app‐based digital mental health interventions targeting distress. As digital technologies become more embedded in children and adolescents' lives, it is imperative to explore how they can support help‐seeking, improve access, address stigma, enhance awareness and contribute to promote mental health and prevent and seek support in times of distress or when experiencing mental health problems (Naslund, 2019).

#### 6.1.7 Early childhood development is the most under‐researched outcome area

During the first years of life, children develop core skills that are crucial to their ability to thrive throughout the life course. This EGM reveals an important research gap: early childhood development constitutes the most under‐researched outcome area of child global mental health, with critical gaps on research investigating behaviour problems during early childhood (0–4). A possible explanation for this gap could be that many early childhood interventions are integrated into maternal mental health interventions and researched within this literature and not independently. In high‐income settings, promotion and prevention interventions such as socio‐emotional learning programmes have shown positive outcomes in later in life such as enhanced wellbeing and adjustment (Eisenberg, 2010; Geldhof, 2011). A recent systematic review of the effects of early parenting interventions on early childhood development outcomes conducted in LMICs found that although trials supported benefits on a wide range of outcomes, they also revealed fading effects over time and inconclusive findings on long‐term impacts (Jeong 2021). A meta‐analysis of parenting interventions conducted in LMICs and encouraging responsive, stimulating, and sensitive caregiver‐child interactions showed small to moderate effects on children's cognitive and language development and motor skills but no effects on socio‐emotional developments, likely due to the lack of research in this area (Zhang, 2021).

#### 6.1.8 Research focuses on the general child population

Another important finding of this EGM is the limited evidence on the effectiveness of these interventions for specific sub‐populations, including groups that tend to report higher prevalence of mental health conditions. It is crucial to understand whether findings from the general child population can be applied to different groups. This would also shed light on whether equality, diversity and inclusion strategies have been successfully applied.

### 6.2 Limitations of the EGM

Due to the large number of studies included in this EGM, a quality appraisal was not conducted. Instead, we collected data on study design as well as the number of participants included in each primary study or the number of studies within each review. Including both primary studies and systematic reviews means that some other primary studies are included within systematic reviews which results in overlap. Although we did not initially establish language constraints throughout the search, we ultimately had to screen out several studies and reviews in languages other than English, Spanish, Portuguese, Chinese, and French. We also excluded grey literature and only included peer‐reviewed reports, reviews, and academic articles. Ultimately, these exclusions could affect the comprehensiveness of this map and result in an over‐representation of studies and reviews among countries where English, Spanish, Portuguese, Chinese, and French are spoken. Due to the large volume of included studies and reviews, only 5% of records were double screened by two separate reviewers on titles and abstracts and only 5% of records were double screened and coded on full text. Efforts were made to secure the quality of independent screening and coding, including random cross‐checks among reviewers, and regular reviewer meetings for rectifying coding standards and methods, reflecting concerns and resolving disagreements by consensus.

We used the machine learning function within Eppi‐Reviewer to help screen out irrelevant studies, which might cause some studies were excluded by this function. There are always spaces for improvements for the accuracy of the machine learning functions in identifying relevant and prevalent studies, and efforts were made to check the accuracy including randomly checking the excluded studies screened out by the machine.

In this EGM, we found a large proportion of studies and reviews focused on treatment interventions in comparison to prevention and promotion interventions. It is important to note, however, that these boundaries are sometimes difficult to draw (Purgato, 2020). This EGM excluded large population‐level programmes such as mental health policies and legal frameworks. Considering the potential of appropriately formulated and implemented policies to improve child and adolescent mental health and that many countries do not possess any policies on mental health (Zhou, 2018), this remains an important area for future research. This EGM focused on the effectiveness of MHPSS interventions on mental health and psychosocial outcomes. However, MHPSS interventions have intended and unintended impact on social outcomes (e.g., strengths of relations within communities, discrimination, violence) and the research is needed within this area (Ubels, 2022). Lastly, due to the large number of studies and reviews identified in this EGM, we did not extract other important criteria such as whether studies measured long‐term effects of interventions or information on who provided the intervention. We encourage reviewers exploring effectiveness findings of these interventions to extract and report on findings of these criteria.

### 6.3 Differences from the protocol

After the publication of the protocol (Sharma, 2022), we revised the resources and added several websites and grey literature sources to our list and removed a few sources. We removed PROSPERO, ClinicalTrials.gov and WHO ICTRP because we had limited time and human resources to contact the authors of ongoing studies for data or publications, these were not providing unique results, and their data were not peer‐reviewed. We removed the Bing search engine because Google already retrieved the test relevant results and considered to be enough. We could not access WHO's Global Health Library; however, we searched Global Index Medicus which is a similar source to WHO's Global Health Library. Although we were able to run all the intended searches in Google Scholar, this source blocked our attempts to export the search results for all the following search strategies. We believe the extent to which we may have missed studies due to this was minimal as we conducted other extensive searches in main databases. We also added CENTRAL to the list of sources as suggested by the peer‐reviewer of the protocol and added HMIC Health Management Information Consortium as a relevant source that was not listed in the protocol.

## 7 AUTHORS' CONCLUSIONS

### 7.1 Implications for research, practice and policy

This EGM aimed to accelerate progress in the field of child and adolescent mental health and psychosocial support by channelling the available evidence and identifying research gaps for action by funders, researchers and policymakers. The body of evidence on this area is complex and it is expanding progressively. However, research on child and adolescent MHPSS interventions is more reactive than proactive, with most evidence focusing on addressing mental health conditions that have already arisen rather than preventing them or promoting mental health.

Further research should investigate the effectiveness of digital mental health interventions for children and adolescents as well as interventions to address the mental health and psychosocial needs of children in humanitarian settings. Research on early childhood MHPSS interventions is urgently needed. MHPSS research for children and adolescents lacks diversity. To better understand, support and promote the mental health of *all* children and adolescents, research should go beyond investigating the effectiveness of interventions for the general population and include sub‐populations which often report higher prevalence of mental health and psychosocial problems and are less likely to have access to mental health. As a next step, wider stakeholder consultations at regional level based on the findings of this EGM can help organisations implement evidence‐based mental health and psychosocial support interventions. These consultations could assist in solving mixed messages about the effectiveness of certain types of interventions for specific mental health outcomes, allow us to do better with the research we already have and address research imbalances.

Mental health and psychosocial support is an institutional priority for the UN and for UNICEF, and is critical to the achievement of the 2030 Sustainable Development Goals. The UNICEF Strategic Plan 2022–2025 identifies MHPSS as a priority area, building upon existing programming through child protection, education and health. Progress on MHPSS is hampered by lack of investment in robust research on which interventions work to improve child and adolescent mental health, especially considering the global burden of disease attributable to mental health disorders (Patel, 2018). Funding for mental health research has been found to be *too inequitable*, with less than 10% of funding being spent in countries that have 90% of global health problems and *too skewed*, with more than 50% devoted to biological research and just about 7% allocated to health services research, clinical and prevention research, respectively (Patel, 2018).

While new donors are emerging and the COVID‐19 pandemic is driving a small uplift in mental health investments for the general population, the limited investment that is allocated for children's, adolescents' and young people's mental health, often only addresses surface‐level factors through reactive interventions rather than proactive programmes. This delivers short‐term wins instead of long‐term change or does not become available until young people have reached a point of crisis. This EGM assists MHPSS practitioners advocate, fund and make child and adolescent MHPSS a global priority.

## CONTRIBUTIONS OF AUTHORS

### Roles and responsibilities

- Content: C. P., R. Y.
- EGM methods: S. B., A. I., C. P.
- Information retrieval: F. S.
- Screening and data extraction: C. P., R. Y., A. I., J. S. M. S.
- Guidance: D. A., S. B.

## DECLARATIONS OF INTEREST

None known.

### Plans for updating the EGM

Once completed, the EGM will be updated yearly, depending on the need of an update (availability of new reviews and primary studies). Regular updates are also subject to availability of funding. If funding is available, UNICEF Innocenti—Global Office of Research and Foresight takes responsibility for updating the review.

## SOURCES OF SUPPORT

Internal sources

- UNICEF Innocenti—Global Office of Research and Foresight, Italy

The funding for this EGM is provided by UNICEF Innocenti—Global Office of Research and Foresight.

External sources

- Not Applicable, Other

Not Applicable.

## Supporting information

Supporting information.
