# Supplementary material for: Child and adolescent mental health and psychosocial support interventions: An evidence and gap map of low‐ and middle‐income countries
Source: Campbell Syst Rev. 2023 Aug 23;19(3):e1349. doi: 10.1002/cl2.1349 (PMC10445093; doi:10.1002/cl2.1349)
Supplement: Supplementary file 1 — Supporting information. [file CL2-19-e1349-s001.docx]

Appendices

## Appendix 1. Definitions

This protocol reflects the terminology that has been used for the State of the World’s Children (SOWC) 2021 report on mental health (UNICEF 2021). As described in the SOWC 2021, the term ‘mental health condition’ describes a wide range of conditions that can vary in severity from mild and temporary to severe and lifelong. The World Health Organization has also used mental health condition in some materials to indicate “mental, neurological and substance use disorders, suicide risk and associated psychosocial, cognitive and intellectual disabilities.” However, the field of mental health uses multiple terms, some of which have more specific meanings. The terms used for negative mental health outcomes collectively include mental disorder, mental ill-health, mental health condition and psychosocial disability. We will be using the term mental health conditions in this EGM. We will also be using the terms mental health and psychosocial well-being to refer to positive mental health outcomes.

## Appendix 2. Search strategies

The search strategies can be accessed online via https://doi.org/10.17605/OSF.IO/WRFN4.

## Appendix 3. PRISMA-S Flowchart


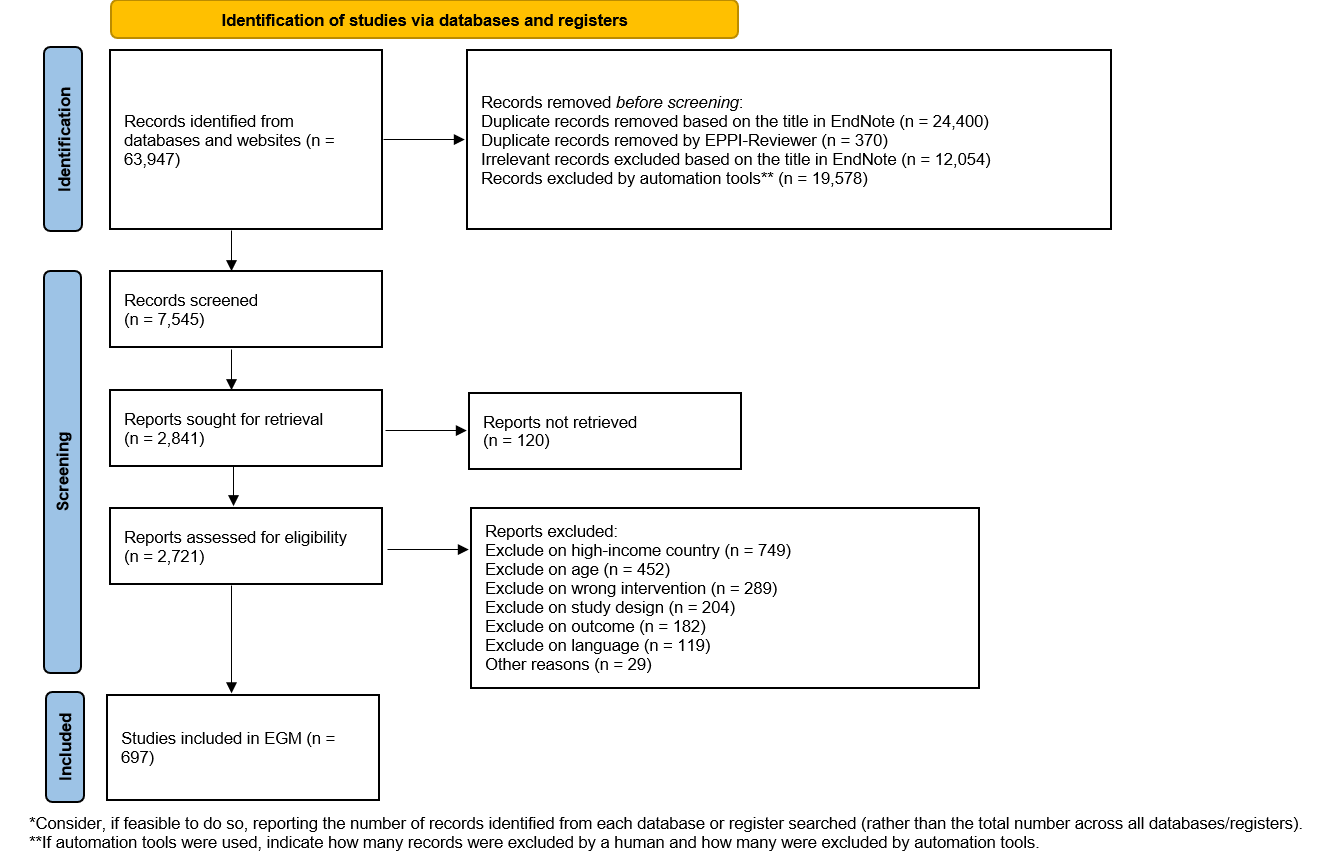


*From:* Page MJ, McKenzie JE, Bossuyt PM, Boutron I, Hoffmann TC, Mulrow CD, et al. The PRISMA 2020 statement: an updated guideline for reporting systematic reviews. BMJ 2021;372: n71. Doi: 10.1136/bmj.n71. For more information, visit: http://www.prisma-statement.org/

## Appendix 4. Screening tool

| General screening questions | No | Yes | Unclear |
| --- | --- | --- | --- |
| • Make quick judgements to exclude documents based on the following screening questions.  • If you cannot exclude, or if you’re unclear after checking against all criteria, then it must be forwarded for abstract screening. | | | |
| Was the study/review published before 2010? |  |  |  |
| If yes, then exclude.  If the study is a key systematic review in the field however, it can be saved in a separate folder for the background section. | | | |
| Does the study/review include children or adolescents (ages 0-19)? |  |  |  |
| If no, then exclude.  There may be some studies that include older age groups as well; as long as it includes anyone aged 0-19 years, it should be included. We will deal with the disaggregation of data for younger ages at the next stage. | | | |
| Is the intervention a biomedical trial of a product, medication or procedure? |  |  |  |
| If yes, then exclude. | | | |
| Does the study/review include focus on or include Low- and Middle-Income Countries (LMICs)? |  |  |  |
| If no, then exclude.  For systematic reviews, there should be at least one primary study from an LMIC included. | | | |
| Does the study/review focus on interventions for mental health promotion, prevention or treatment? |  |  |  |
| If no, then exclude. | | | |
| Does the article include any of the following study designs?  Randomized controlled trials, quasi‐experimental studies, mixed-methods studies, systematic reviews or meta-analyses. |  |  |  |
| If no, then exclude. | | | |
